# In situ constructing atomic interface in ruthenium-based amorphous hybrid-structure towards solar hydrogen evolution

**DOI:** 10.1038/s41467-023-37451-7

**Published:** 2023-03-28

**Authors:** Dong Liu, Tao Ding, Lifeng Wang, Huijuan Zhang, Li Xu, Beibei Pang, Xiaokang Liu, Huijuan Wang, Junhui Wang, Kaifeng Wu, Tao Yao

**Affiliations:** 1 National Synchrotron Radiation Laboratory, University of Science and Technology of China, Hefei, 230029 P. R. China; 2 State Key Laboratory of Molecular Reaction Dynamics and Dynamics Research Center for Energy and Environmental Materials, Dalian Institute of Chemical Physics, Chinese Academy of Sciences, Dalian, Liaoning 116023 P. R. China; 3 Experimental Center of Engineering and Materials Science, University of Science and Technology of China, Hefei, 230026 P. R. China

**Keywords:** Photocatalysis, Artificial photosynthesis, Characterization and analytical techniques

## Abstract

The rational steering and construction of efficient and stable atomic interfaces is highly desirable but rather challenging in solar energy conversion. Here, we report an in-situ oxygen impregnation strategy to build abundant atomic interfaces composed of homogeneous Ru and RuO_x_ amorphous hybrid-mixture with ultrafast charge transfer, for solar hydrogen evolution with sacrificial agent free. Via in-situ synchrotron X-ray absorption and photoelectron spectroscopies, we can precisely track and identify the gradual formation of atomic interfaces towards homogeneous Ru-RuO_x_ hybrid-structure at the atomic level. Benefiting from the abundant interfaces, the amorphous RuO_x_ sites can intrinsically trap the photoexcited hole within an ultrafast process (<100 fs), and the amorphous Ru sites enable subsequent electron transfer (~1.73 ps). Hence, this hybrid-structure triggers long-lived charge-separated states, and results in a high hydrogen evolution rate of 60.8 μmol·h^−1^. This design integrating the two sites fulfilled each half-reaction in a single hybrid-structure suggests potential guidelines towards efficient artificial photosynthesis.

## Abstract (web summary)

Solar-driven hydrogen evolution coupled with organic synthesis is important but challenging. Here, the authors report an in-situ oxygen impregnation strategy to build a ruthenium-based amorphous hybrid-mixture with abundant atomic interfaces and show efficient hydrogen evolution with small molecule oxidation.

## Introduction

Solar-driven H_2_ evolution from water using nanocatalysts is a fascinating energy solution and has been intensively studied^1–5^. To the best of our knowledge, most of the pioneering H_2_ evolution systems were based on a further addition of sacrificial agents such as triethanolamine (TEOA), triethylamine (TEA), and ethyl acetate (EAA) which were used to scavenge holes to suppress undesired electron-hole recombination and lengthen the lifetime of charge-separated states for the efficient H_2_ evolution^6–9^. For those that do not involve sacrificial agents, they generally require the deposition of noble metal co-catalysts^10,11^. Up to now, rational constructing photocatalytic H_2_ evolution systems in the absence of any sacrificial agents is an essential but still challenging issue^4,12^. Previous spectroscopic studies suggested that the hole transfer to the electron donor is the key efficiency-limiting step towards achieving efficient H_2_ generation^13–15^. Therefore, exploring suitable and efficient interface with ultrafast hole trapping ability provides the possibility to realize the efficient H_2_ evolution^16–19^. The common systems additionally integrate with ruthenium-based molecules or ruthenium oxide to trap holes due to the variable valence of ruthenium^12,20^, while it has rarely been reported on only one system that can simultaneously trap hole and catalyze H_2_ evolution.

Currently, most of the bifunctional design is to deposit co-catalysts or to prepare heterojunction, where only single interface between two components is formed. As a result, the separation of photo-electrons/holes, decoupling of the H_2_/O_2_ evolution reactions, and the migration of reaction intermediates, occurred on such single interface could be greatly limited. Hence, more interfaces be made would be helpful for promoting the whole reaction efficiency. On the other hand, distinctive from crystalline nanocrystals, amorphous nanoparticles exhibit unique physical and chemical properties, such as abundant defects, unsaturated coordination sites, and flexible structure^21–23^, which strongly enables an outstanding photo/electrocatalytic performance in multiple circumstances^24–26^, but has rarely been reported in photocatalytic applications^27^. Moreover, the synthesis of amorphous metal nanomaterials under mild conditions remains to be a big challenge and hinders its application. This is mainly due to the fact that metal atoms tend to integrate together by unoriented metal bonds, and then form crystals via translational motion^28^, and the case is more severe for noble metal-based amorphous structures as they propose stronger metallic bonds compared with transitional metals^29,30^. Fortunately, taking advantage of the preselected precursor ruthenium carbonyl (Ru_3_(CO)_12_) with intermolecular forces and its strong interaction with graphitic carbon nitrides (g-C_3_N_4_) support, it would suppress crystallization and achieve successful preparation of amorphous Ru nanoparticles. Therefore, if the Ru sites for electron-triggering H_2_ evolution and the RuO_x_ sites for hole trapping can be effectively integrated at atomic scale with multiple heterointerfaces, it will be promising to achieve high-efficiency photocatalytic H_2_ evolution.

Herein, via the oxygen impregnation strategy, we designed and successfully synthesized an amorphous Ru-RuO_x_ hybrid-structure decorated on g-C_3_N_4_ support, in which the amorphous Ru and RuO_x_ species were homogeneously mixed at the atomic scale in a nanoparticle with multi-heterointerface. The time-resolved photoluminescence (PL) and ultrafast transient absorption (TA) spectroscopies verify this effective design, revealing that the amorphous RuO_x_ sites can intrinsically trap the photoexcited hole in an ultrafast process of <100 fs and the amorphous Ru sites enable an efficient electron transfer of 1.73 ps which results in a long-lived charge-separated state for reaction. As a result, the amorphous Ru sites were used as the H_2_-evolving component for coupling with another amorphous RuO_x_ sites as the holes trapping component, achieving the efficient sacrificial agent-free photocatalytic H_2_ evolution of 60.8 μmol·h^−1^. Besides, coupled with furfuryl alcohol oxidation reaction, the bifunctional structure with muti-heterointerfaces exhibited stable H_2_ and furfural evolution activities. Moreover, we identified the oxygen impregnation process by in-situ X-ray absorption fine structure (XAFS) and X-ray photoelectron spectroscopy (XPS) depth profiling techniques. Our results not only provide a promising system for efficient photocatalytic H_2_ evolution, but address a potential strategy for the construction of atomic multi-interface for boosting other coupling catalytic reactions.

## Results and discussion

### Synthesis and structural characterization

**Fig. 1:**
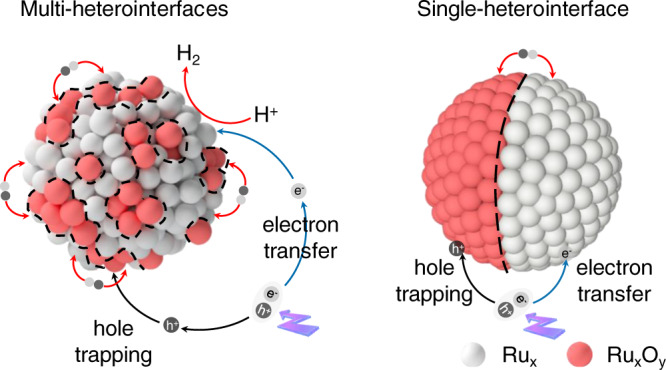
Schematic illustration of the catalytic system. Schematic illustration of the solar-driven H_2_ evolution system on Ru-RuO_x_/C_3_N_4_ with multiple and single hetero-interface.

**Fig. 2:**
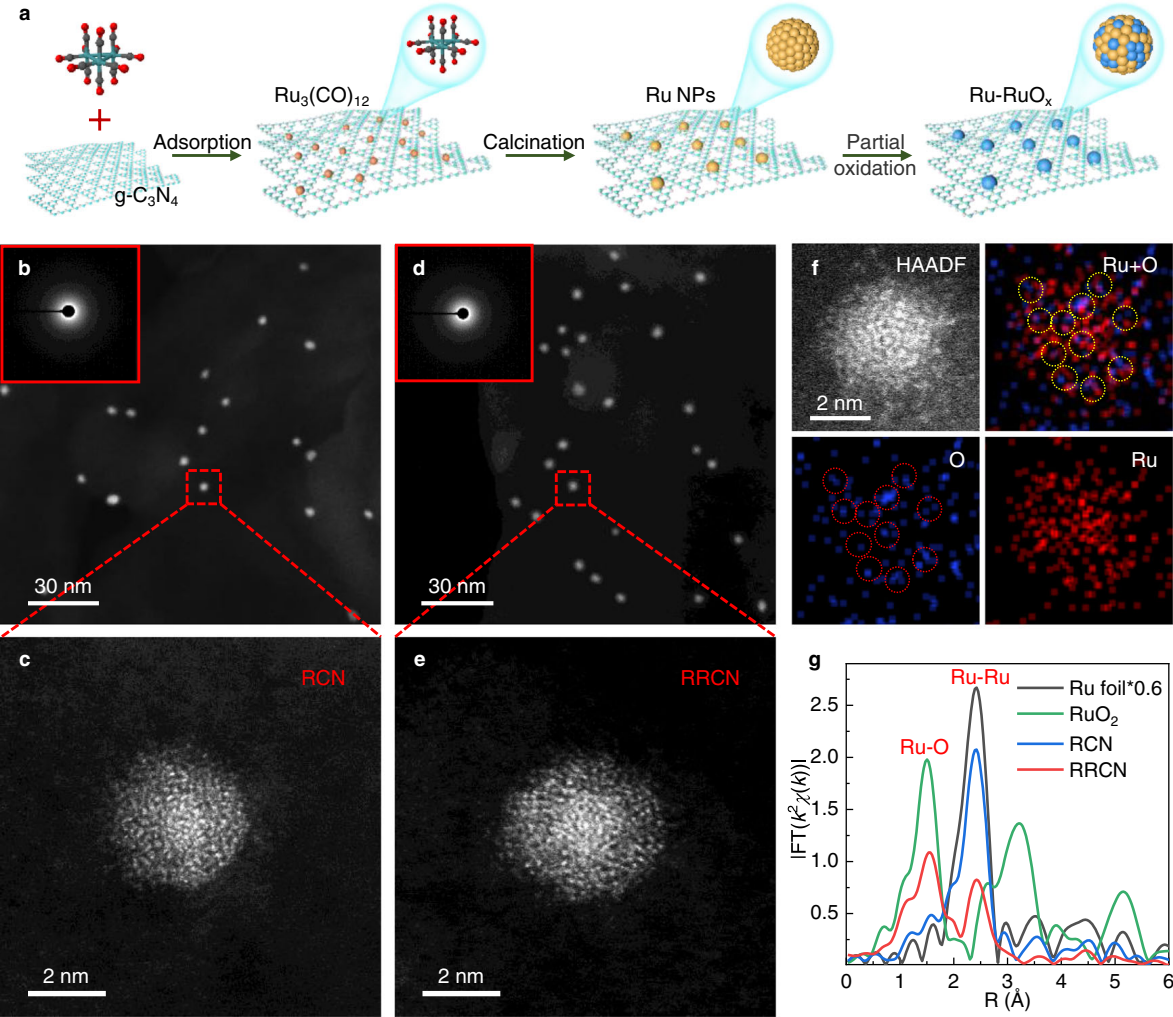
Preparation and characterization of the photocatalyst. **a** Schematic illustration of the preparation strategy for RCN and RRCN. **b**–**e** HAADF-STEM, SEAD (inset), and high-resolution AC-HAADF-STEM images of RCN (**b**, **c**) and RRCN (**d**, **e**), respectively. **f** AC-HAADF-STEM image and near atomic resolution mappings of RRCN. **g** *k*^2^-weighted Fourier transform (FT) Ru *K*-edge EXAFS spectra for RCN, RRCN and Ru foil and RuO_2_ (no phase correction).

The designing of the photocatalytic H_2_ evolution system was schematically illustrated in Fig. 1. Through the strategy of in-situ oxygen impregnation, we homogeneously integrated the amorphous Ru and RuO_x_ species into a hybrid-nanoparticle at the atomic scale, and thus creating multiple heterointerfaces. The amorphous RuO_x_ sites generated in situ were designed for hole trapping, while the amorphous Ru sites were designed as the electron acceptor for catalytic reaction. As such, compared with the traditional heterojunction with single heterointerface and few contact surfaces, the photogenerated electrons and holes from g-C_3_N_4_ support can be effectively separated, and the reduction/oxidation reactions can be decoupled spatially, on such atomically homogeneous Ru-RuO_x_ amorphous hybrid-structure. The preparation of amorphous Ru and Ru-RuO_x_ nanoparticles anchored on g-C_3_N_4_, named RCN and RRCN, respectively, was represented in Fig. 2a (for details see “Methods”). Starting from bulk g-C_3_N_4_ obtained by thermal polymerization of urea, the RCN photocatalyst was synthesized by calcinating ruthenium carbonyl (Ru_3_(CO)_12_) which was fully adsorbed on ultra-thin g-C_3_N_4_ nanosheets (Supplementary Fig. 1). Subsequently with a partial oxidation process, the RRCN as a H_2_ evolution photocatalyst was successfully constructed. The Ru content for RCN and RRCN was determined to be ~1.5 wt% according to the inductively coupled plasma optical emission spectrometry (ICP-OES) analysis.

In order to examine the morphology and structure of Ru species in the photocatalysts, transmission electron microscopy (TEM) was first carried out. As shown in Fig. 2b and Supplementary Fig. 2, the Ru nanoparticles in the RCN sample with an average diameter of 3.8 nm were uniformly dispersed on the g-C_3_N_4_ nanosheets (CN). As shown in the Aberration-corrected high-angle annular dark-field scanning transmission electron microscopy (AC-HAADF-STEM) image and high-resolution transmission electron microscopy (HRTEM) image (Fig. 2c and Supplementary Fig. 2), the Ru nanoparticles exhibit the disordered atomic structure, confirming the amorphous feature. Similarly, the dispersive morphology and amorphous nature remained unchanged for the partially oxidized Ru-RuO_x_ nanoparticles, except that the average particle diameter increased to 4.4 nm (Fig. 2d, e and Supplementary Fig. 3) which is possibly resulted from the impregnation of oxygen atoms into the amorphous Ru particles. In addition, energy-dispersive X-ray spectroscopic (EDS) mapping analysis revealed the homogeneous dispersion of Ru species in the RRCN and RCN samples (Supplementary Figs. 4 and 5). It is noteworthy that, compared to RCN, the EDS mapping distribution of the oxygen element in RRCN was well consistent with Ru nanoparticles, thus manifesting the formation of RuO_x_ component. Besides, the near atomic resolution mapping of the RRCN sample in Fig. 2f showed that the O atoms were randomly distributed in the amorphous Ru-RuO_x_ hybrid-particle, which indicated that the hybrid-particles existed in the form of multi-Ru-RuO_x_ heterointerface rather than core-shell structure^31^. Furthermore, selected area electron diffraction (SEAD) and X-ray powder diffraction (XRD) tests were conducted to further confirm the amorphous feature of Ru species in RCN and RRCN. As shown in the SEAD patterns (the inserts in Fig. 2b, d), no diffraction spot or diffraction ring of Ru/RuO_2_ can be found except the only diffraction ring indexed to the (002) crystal plane of CN^32^. Meanwhile, neither Ru nor RuO_2_ diffraction peak were observed in the XRD patterns (Supplementary Fig. 6). However, once RuCl_3_ was served as the metal precursor instead of Ru_3_(CO)_12_, both Ru and Ru-RuO_x_ particles existed as crystalline structure under the same synthetic procedure (Supplementary Figs. 7 and 8). This is likely due to that the Ru^3+^ ions adsorbed on the support are prone to migration, nucleation, and growth during the annealing process lacking of intermolecular interaction, which led to the formation of nanocrystalline Ru particles.

To further identify the local structures of the nanoparticles, we performed synchrotron radiation X-ray absorption near edge structure (XANES) and extended X-ray absorption fine structure (EXAFS) tests at the Ru *K*-edge. As shown in the XANES spectra (Supplementary Fig. 9), compared with Ru foil, the absorption edges of RRCN shifted to the higher energy side, along with an increase of the white line-peak intensity, meaning that the oxidation states of Ru increased^33,34^ which was attributed to the generation of RuO_x_. Furthermore, Fig. 2g shows the corresponding Fourier transformed *k*^2^-weighted EXAFS spectra for RCN and RRCN. Two dominant peaks of the RRCN sample at approximate 1.56 Å and 2.40 Å can be assigned to the Ru-O and Ru-Ru coordination^35^, suggesting the formation of RuO_x_ species due to the impregnation of oxygen atoms into Ru nanoparticles. Besides, the position of Ru-O coordination peak in RRCN is slightly positive than that in RuO_2_ (1.53 Å), which demonstrated the volume expansion of amorphous Ru-RuO_x_ particles compared to the crystalline RuO_2_ and was in good agreement with the above electron microscopy results.

### In situ tracking the controlled oxygen impregnation process

**Fig. 3:**
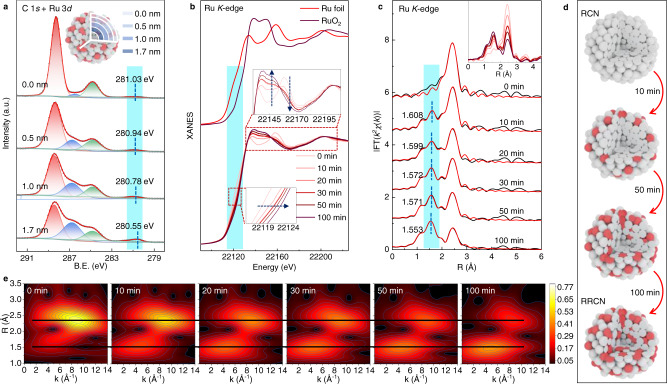
In situ oxygen impregnation process. **a** High-resolution XPS spectra at different sputtering depths for Ru 3*d* of the RRCN sample. **b** In-situ XANES spectra recorded at the Ru *K*-edge of RRCN at different oxidation time during the heat process, and the XANES data of the referenced standards of Ru foil and RuO_2_. Inset, Magnified absorption edge and white-line peak of XANES region. **c** Least-squares curve-fitting analysis of in-situ EXAFS spectra at the Ru *K*-edge. Inset, In-situ XANES spectra recorded at the Ru *K*-edge of RRCN at different oxidation time during the heat process. **d** Schematic illustration of the oxygen insertion process during the formation of RRCN. **e** Wavelet analysis of the RRCN sample during in-situ EXAFS states.

To further understand the existence states of Ru and RuO_x_ species and their dynamic evolutions during the oxygen insertion process, X-ray photoelectron spectroscopy (XPS) depth profiling with ion beam etching was carried out first. The RRCN sample was deposited on silicon wafer and treated with argon ion beam sputtering which can remove 10 nm surface samples per minute. As shown in the XPS spectra of Ru 3*d* orbital (Fig. 3a), the peak located at 281.03 eV in the pristine RRCN sample can be assigned to the Ru 3*d*_5/2_ state of oxidated Ru^*δ*+^. With the sputtering depth increased to ~1.7 nm gradually, the Ru 3*d*_5/2_ peak shifted to 280.55 eV, suggesting the decreasing of the oxidation state of Ru^36^. In addition, as the oxygen impregnation depth increases, the resistance goes higher, resulting in the higher oxidation state of Ru on the surface of the hybrid Ru-RuO_x_ nanoparticles than that in the interior. According to the XPS depth profiling, the inset in Fig. 3a illustrated the existence state of Ru-RuO_x_ nanoparticles in the RRCN sample, in which the Ru and RuO_x_ clusters are randomly distributed to form the uniquely homogeneous heterostructures. Different from the separated Ru and RuO_2_ nanoparticles, a large number of Ru/RuO_x_ heterointerfaces in RRCN could enable the rapid separation/slow recombination of the photoexcited carriers and participation in chemical reactions, thus promoted the photocatalytic H_2_ evolution.

Accompanied by the evolution of local geometric structure, the composition and electronic structural changes of the nanoparticles during the partial oxidation process can be revealed by the in situ XAFS results at the Ru *K*-edge. The in situ XAFS experiment was conducted in a homemade cell under an air atmosphere of 200 °C. Beginning with the RCN sample, a series of XAFS spectra were collected in different oxidation time (*T*_t_, t = 0, 10, 20, 30, 50, 100 min). The XANES results in Fig. 3b showed that as the oxidation time increased, the absorption edge slightly shifted to the higher energy position, indicating the increase of Ru oxidation state due to the insertion of oxygen atoms. As reflected by the absorption edges of XANES spectra for Ru foil and RuO_2_ standards, the Ru valence states in all of the samples lie between 0 and +4 (Fig. 3b and Supplementary Fig. 10). In addition, after comparing with the Ru foil and RuO_2_ references, we can find the sequential evolution of the white line peaks of the RRCN-*T*_t_ spectra, implying that the oxygen is inserted into the Ru nanoparticles to form RuO_x_.

Next, the local structural evolution of the atomically homogeneous Ru-RuO_x_ amorphous hybrid-nanoparticles was identified by EXAFS. Figure 3c exhibited the typical Fourier transformed (FT) *k*^2^-weighted EXAFS spectra for different oxidation times, together with the referenced samples (Supplementary Fig. 11). With respect to the T_0_ spectrum, the dominant peak at approximately 2.43 Å can be assigned to the metallic Ru-Ru coordination. As the high-temperature oxidation process started, a fresh peak at approximately 1.61 Å (T_10_) appeared which can be assigned to the Ru-O coordination. As the reaction time increased, the intensity of Ru-Ru peak at 2.43 Å gradually decreased, accomplished by the increase of the Ru-O coordination peak. This result implies that the Ru nanoparticles transforms into Ru-RuO_x_ by degrees, which well coincides with the XANES results. Figure 3d illustrated the oxygen impregnation process during the formation of RRCN. With the continuous oxygen impregnation, the position of Ru-O coordination peak shortened to ~1.55 Å. Quantitative structure information can be obtained by least-squares EXAFS curve-fitting analysis (Fig. 3d and Supplementary Table 1), and the corresponding Re(*k*^2^*χ*(*k*)) oscillation and fitting curves are exhibited in Supplementary Figs. 12 and 13. For the T_0_ curve, the best fitting result showed coordination number (CN) of 6.6 for Ru-Ru, lower than that of Ru foil which is 12. This strongly suggested that the Ru nanoparticles existed in a small size and amorphous state, resulted in high surface atomic ratio and a large number of unsaturated coordination sites. Interestingly, the CNs of Ru-Ru and Ru-O gradually decreased from 6.6 to 3.1 and increased from 0 to 3.4, respectively, along with the continuous impregnation of oxygen atoms, reflecting that the amount of RuO_x_ component in the homogeneous Ru-RuO_x_ heterostructures can be well tuned. In addition, the controlled experiments of RRCN-cry-*T*_t_ (t = 30, 50, 100) samples were conducted under the same condition (Supplementary Fig. 14). It can be found that the crystalline Ru particles transformed into RuO_x_ immediately as the high-temperature oxidation process initiated, validating that the amorphous nanoparticles had much higher stability than the crystalline ones.

The wavelet transform (WT) of EXAFS in k-space was employed to confirm the coordination information on the Ru site. The WT contour plots with optimum resolution were drawn based on the Morlet wavelet. The WT analysis of RRCN-*T*_t_ spectra showed two prominent maximum values at 4.8 Å^−1^ and 8.9 Å^−1^ (Fig. 3e and Supplementary Fig. 15), which are assigned to the Ru-O and Ru-Ru scattering paths^37^. Similar to EXAFS results, the bond length of Ru-O shortened, and the scattering intensity of Ru-O increased little by little. These detailed WT-EXAFS analyses and the in-situ XAFS characterizations shed light on the dynamic oxygen insertion process in the formation of hybrid Ru-RuO_x_ nanoparticles, which is potentially conductive to the atomic-precisely design and synthesis of photo/electrocatalyst.

### Optical and semiconductor properties

**Fig. 4:**
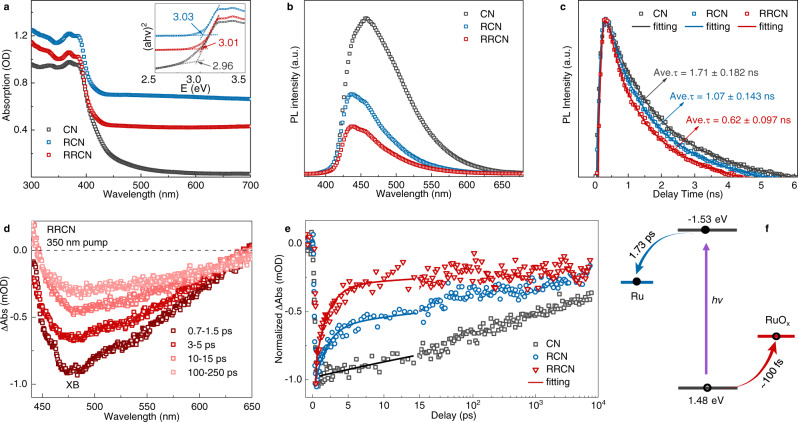
Optical and charge-transfer properties. **a** UV-vis diffuse reflectance spectroscopy of CN, RCN, and RRCN. Insert, Tauc plot of the diffuse reflectance data in (**a**). **b** Steady-state PL spectra at 298 K. **c** Time-resolved PL kinetics at the corresponding steady-state emission peaks in (**c**) at 298 K. **d** TA spectra of RRCN probed at the indicated time delays following excitation by a 350 nm pulse. **e** TA kinetics probed at the XB center (∼500 nm; open symbols). **f** Physical picture of charge transfer.

The ultraviolet-visible diffuse reflectance spectra (DRS) were performed to verify the optical properties of the synthesized RCN and RRCN samples. As shown in the UV-vis DRS of the RCN and RRCN samples (Fig. 4a), they inherited the photophysical features of C_3_N_4_. All the samples display similar bandgap (nearly 2.96 eV, inset in Fig. 4a) and strong absorption in the range below 435 nm dominated by the C_3_N_4_ nanosheets, indicating their similar light absorption behavior^38^. Notably, the RCN and RRCN samples showed a strong and broadband enhanced absorption tail from 440 nm to 800 nm compared with pristine C_3_N_4_. This feature cannot arise from scattering and can be attributed to the interband transition in Ru/RuO_x_ nanoparticles, which is in agreement with the color change of samples from bright yellow (C_3_N_4_) to dark blue (RCN) and gray blue (RRCN) (Supplementary Fig. 16).

To further investigate the efficient electron-hole separation between the Ru-RuO_x_ nanoparticles and C_3_N_4_ support, the steady-state PL measurements were employed upon excitation with 350 nm light. The PL peaks of all samples were at ~456 nm, corresponding to a Stokes shift of ~0.1 eV of C_3_N_4_. As shown in Fig. 4b, the PL quenching efficiency increased from ~51.2% for RCN to ~72.1% for RRCN, reflecting the electron and sequential hole transfer to the Ru/RuO_x_ component on the basis of the schematic energy level alignment^39^. Moreover, time-resolved PL spectra recorded at the corresponding steady-state emission peaks were also performed (Fig. 4c). Compared with C_3_N_4_ nanosheet, the decay of PL features was accelerated for the RCN and RRCN samples, which could be attributed to the amorphous Ru nanostructure and atomically homogeneous Ru-RuO_x_ amorphous hybrid-structure with multi-heterointerfaces. By fitting the PL kinetics in Fig. 4c, we extracted the averaged lifetime of 1.71 ± 0.18 ns, 1.07 ± 0.14 ns, 0.62 ± 0.1 ns for free C_3_N_4_, RCN, and RRCN, respectively, which was also consistent with the static PL spectra and further confirmed the charge transfer from photoexcited C_3_N_4_ to the amorphous nanoparticles. In order to reveal the charge transfer of the as-synthesized photocatalysts, ultrafast TA spectroscopy with a femtosecond pump-probe configuration was carried out. Briefly, a pump pulse at 350 nm was used to selectively pump the C_3_N_4_, and the induced absorption changes were monitored by a white light continuum probe pulse, which provides a complete record of electron transfer, hole trapping, and recombination events. Figure 4d showed the typical TA spectra of the RRCN sample at indicated delays. The spectral features are dominantly contributed by C_3_N_4_, including an exciton bleach (XB) feature at ∼475 nm. The XB feature can be assigned to state-filling-induced bleach by photogenerated excitons (CB electrons and VB holes)^40,41^.

To further unveil the excited state dynamics of these photocatalysts, their TA kinetics of XB at 500 nm were compared. Notably, the decay tendency of the XB and the time-resolved PL (Fig. 4c) were in excellent agreement with each other for the CN, RCN, and RRCN samples. Considering that the charge transfer process almost accomplished within 15 ps for the RRCN and RCN samples, all the kinetics traces can be fitted (Fig. 4e and Supplementary Fig. 17) in this timescale. The decay of XB kinetics for the free C_3_N_4_ sample can be well-fitted using a mono-exponential decay function, resulting from the inherent photogenerated charge recombination. Compared to the free C_3_N_4_ sample, the fitting result of the RCN sample showed an electron transfer process with a time constant of 1.73 ps, owning to the electron transfer of C_3_N_4_ to amorphous Ru nanoparticles. Moreover, the RRCN sample possessed an additional channel for charge transfer. Due to the hole transfer ability of ruthenium-based molecules or ruthenium oxide^12^ and the instrument response function (IRF) of the TA setup (~100 fs), it is strongly suggested that the hole capturing time from the VB of C_3_N_4_ to amorphous RuO_x_ component is <100 fs. Such femtosecond hole capturing and the picosecond electron transfer enable an extremely long-lived charge-separated states (recombination between an electron in the Ru site and a hole captured by the RuO_x_ site), exceeding the time window of 8 ns for TA test (Fig. 4e and Supplementary Fig. 17). On the basis of the UPS (Supplementary Fig. 18), TA and time-resolved PL spectroscopy results, a unified physical picture for the charger transfer models of the RRCN sample is summarized in Fig. 4f and Supplementary Fig. 19. Under irradiation light, taking the advantage of amorphous structure and atomically homogeneous multi-heterointerfaces, the amorphous RuO_x_ component traps the photogenerated hole in an ultrafast process of <100 fs and the amorphous Ru site enables an ultrafast electron transfer process of 1.73 ps. As a result, a long-lived charge-separated state is formed, which is long due to the reduced electron-hole wave function overlap. It is beneficial to the efficient accumulation of the transferred electrons into Ru site, possibly accounting for the significantly improved photocatalytic activity. Due to its efficient charge transfer and fast switching of reactive species, this special atomically homogeneous Ru-RuO_x_ hybrid-structure with multiple heterointerfaces will play an important role in photo/electrocatalytic applications.

### Evaluation of photocatalytic performance

**Fig. 5:**
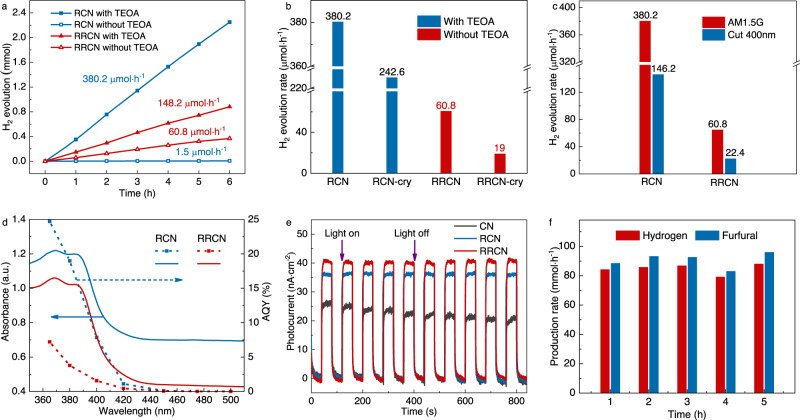
Catalytic performance of RCN and RRCN. **a** Typical time course of H_2_ productions under light irradiation (*λ* ≥ 350 nm) for CN, RCN (left, with 15 vol % TEOA as sacrificial agent), and RRCN (right, without sacrificial agent). **b** Comparative presentation of the H_2_ evolution rates. **c** Photocatalytic H_2_ evolution activities under simulated sunlight (*λ* ≥ 350 nm) and visible (*λ* ≥ 400 nm) light irradiation. **d** AQYs and H_2_ evolution rates of RCN and RRCN under irradiation with light of different wavelengths. **e** *I*–*t* curves of various catalysts with and without light at 0.3 V (*vs*. Ag/AgCl). **f** H_2_ productions and the furfuryl alcohol oxidation to furfural performance for the RRCN sample.

The photocatalytic H_2_ evolution performance of the as-obtained catalysts was evaluated in a closed cylinder Pyrex glass container (for details see “Methods”). The photocatalytic experiment of the RCN sample was conducted in neutral water in the presence of TEOA (15 vol%) as sacrificial reagent under simulated solar illumination conditions (AM 1.5G filter), while that of the RRCN sample was conducted without any sacrificial reagents. Figure 5a illustrated the typical H_2_ evolution rate of the RCN and RRCN samples, respectively. Compared with the pristine C_3_N_4_ (1.8 μmol·h^−1^) which had poor photocatalytic efficiency, the H_2_ evolution rate of amorphous Ru nanoparticles modified RCN remarkably increased to 380.2 μmol·h^−1^, ~200-fold higher than that of pristine C_3_N_4_. In addition, under the condition of pure water without any sacrificial reagents, co-catalysts, or other additives, the optimized oxygen inserted RRCN sample exhibited exciting performance with the H_2_ evolution rate up to 60.8 μmol·h^−1^. On the basis of the photocatalytic tests, we deduced that Ru component in Ru-RuO_x_ served as the active sites for H_2_ evolution, while the RuO_x_ worked as the hole trapping agent. Besides, the controlled experiments which modified Ru nanocrystalline particles were also performed under the same condition. As shown in Fig. 5b, the RCN and RRCN samples modified by amorphous Ru nanoparticles exhibited better photocatalytic activities than the Ru nanocrystalline modified RCN-cry and RRCN-cry samples, with 1.5 and 3.2-fold higher H_2_-evolution rates, respectively. Distinctive from Ru nanocrystals with lattice periodicity, the specific disordered atomic structure of amorphous Ru nanoparticles endowed them with highly chemical homogeneity, abundant defects, and unsaturated coordination sites, thus achieving better photocatalytic H_2_ evolution activity.

Considering the limitation of C_3_N_4_ semiconductor on near ultraviolet light and the demand for the use of visible light in the development of new energy, we also evaluated the performance of the designed RCN and RRCN catalysts under visible light (*λ* ≥ 400 nm). As exhibited in Fig. 5c, in the visible range, the wide band gap leaded to a decrease of photogenerated excitons, resulting in a sharply drop in the H_2_ evolution rate of RCN and RRCN to 146.2 μmol·h^−1^ and 22.4 μmol·h^−1^, respectively. Figure 5d and Supplementary Fig. 20 showed the wavelength-dependent apparent quantum yields (AQYs) and H_2_ evolution rate of RCN and RRCN under irradiation light. Specifically, the obtained RRCN achieved a 7.4% quantum efficiency at 380 nm, 3.1% at 400 nm, and 0.8% at 420 nm. Unfortunately, the H_2_ evolution rate of the RRCN and RCN samples in the visible light range is significantly reduced due to the weak absorption of g-C_3_N_4_ support to visible light^42^. Despite of the relatively reduced performance in the visible light range, our current work provides a very promising system based on the atomically homogeneous Ru-RuO_x_ amorphous hybrid-structure for the separation and utilizing of photogenerated carriers. If the band structure of light-absorbing centers could be tuned to further improve the photoresponse, higher AQYs performance would be achieved under visible light.

To further investigate the charge separation characters of the photocatalysts under working condition, photoelectrochemical (PEC) tests were performed. As shown in Fig. 5e, the photocurrent-time curves indicated that the photocurrents were enhanced for the RCN and RRCN samples compared to the pristine C_3_N_4_, proving that the loading of amorphous Ru and Ru-RuO_x_ nanoparticles boosts the separation of photoexcited e^−^–h^+^ pairs. The construction of the amorphous Ru/Ru-RuO_x_ nanoparticles and C_3_N_4_ can accelerate the carriers transporting from C_3_N_4_ support to the nanoparticles to participate in the redox reaction. Moreover, according to the electrochemical impedance spectroscopy (EIS) (Supplementary Fig. 21), compared with the light off condition, all of the RCN, RRCN and pristine C_3_N_4_ samples possessed smaller radii under light irradiation. The RRCN sample displayed the smallest radius than that of the RCN and pristine C_3_N_4_ samples, demonstrating the lowest charge-transfer resistance and thus achieved efficient charge separation^43^, which was highly consistent with the above photocatalytic and spectroscopy results.

In order to investigate the stability and underlying reaction mechanism of RRCN catalysts, we performed recycling experiments and characterized the samples after the reaction. As shown in Supplementary Fig. 22, the RRCN sample can stably produce H_2_ at the beginning of the reaction, but it is rapidly inactivated after a period of time. The morphology of the RRCN catalyst shown in Supplementary Fig. 23 exhibits a similar amorphous structure to the pristine one. While as can be seen from the XPS results of Ru 3*d* orbits (Supplementary Fig. 24), the valence state of Ru after the hydrogen evolution reaction (RRCN-AR) increases significantly which might be due to the hole oxidation of Ru^0^ and the lower value Ru^*α*+^O_x_ species to the higher value Ru^*δ*+^ in the H_2_ evolution reaction. Similar results can be seen in the XANES and EXAFS spectra in Supplementary Fig. 25, the absorption edge and white line peak of RRCN-AR sample were significantly shifted to the right and up, respectively, and the Ru-Ru scattering peaks in R space almost disappeared, indicating that the valence state of Ru in RRCN increased significantly. Consequently, we suspect that at the beginning of the catalytic reaction, both the Ru^0^ component and Ru^*α*+^O_x_ component exist in the RRCN sample, and the Ru^0^ component is used as hydrogen evolution sites to reduce protons to produce hydrogen, and the Ru^*α*+^O_x_ component with lower value Ru^α+^ captures and consumes holes due to the variable valence state of Ru^*δ*+^ (+2, +3, +4, +6), and its own oxidation occurs. When the reaction time is prolonged, the Ru species and the functional groups such as -NH_2_ in g-C_3_N_4_ carrier are oxidized, the ability of Ru^*δ*+^O_x_ with higher value Ru^*δ*+^ consuming holes is greatly reduced, so that the H_2_ evolution of the whole system cannot be realized. To further highlight the effectiveness of our designed RRCN catalyst with multi-heterointerfaces, we applied it to the photocatalytic H_2_ evolution and furfuryl alcohol oxidation coupling system. The results in Fig. 5f and Supplementary Fig. 26 showed that the RRCN catalyst exhibited high activity with a H_2_ evolution rate of 84.8 μmol·h^−1^. Meanwhile, the recycling experiment in Supplementary Fig. 27 conducted that the furfuryl alcohol consumed photoexcited holes in time, which greatly enhanced the stability of the system. Moreover, both the XPS and XAFS results in Supplementary Figs. 24 and 25 indicated that the valence state of Ru rarely changed during the stability test, heralding multi-heterointerfaces system as candidates for artificial photosynthesis.

In summary, compared with the traditional photocatalytic H_2_ evolution catalysts that require hole sacrificial agents or reaction sites, we have developed a novel strategy to in-situ construct an integrated photocatalyst composed of atomically homogeneous Ru-RuO_x_ amorphous hybrid-structure with multi-heterointerfaces supported on g-C_3_N_4_ nanosheet which could achieve femtosecond hole trapping, therefore, exhibited exceptional activity for efficient solar-driven H_2_ evolution and artificial photosynthesis. Our study demonstrated that under irradiation light, the amorphous RuO_x_ and Ru sites can instantly trap the photogenerated holes and enable an efficient electron transfer, respectively, thus resulting in an extremely long-lived charge-separated state for reaction. Besides, combined the oxygen impregnation strategy, for the first time, we identified the entry and diffusion of oxygen in the formation of Ru-RuO_x_ hybrid-structure at the atomic scale by in-situ XAFS technique. These findings not only provide an efficient artificial photosynthesis system based on the amorphous Ru-RuO_x_ hybrid-structure with the ultrafast hole trapping ability, but also unravel the essential structural evolution that can guide the rational design of amorphous heterogeneous catalysts.

## Methods

### Chemicals

All the chemicals were used without further purification. Urea (CH_4_N_2_O), methanol (CH_3_OH), triethanolamine (TEOA), were acquired from Sinopharm Chemical Reagent Co., Ltd. Ruthenium Carbonyl (Ru_3_(CO)_12_), ruthenium (III) chloride (RuCl_3_), were acquired from Sigma-Aldrich.

### Synthesis of g-C_3_N_4_ (CN)

The carbon nitride (g-C_3_N_4_) was first synthesized as a support. Briefly, a certain amount of urea was put into a covered crucible and then heated to 600 °C for 2 h in a muffle furnace with a heating rate of 5 °C/min. After cooling to room temperature, the obtained flavescent powder was washed with deionized water to eliminate the dust static electricity and remove impurities, whereafter, collected by filtration, and finally dried under vacuum at 60 °C.

### Synthesis of Ru NPs/C_3_N_4_ (RCN)

In a typical synthesis, the g-C_3_N_4_ powder (300 mg) was dispersed in 150 mL MeOH and sonicated for 12 h. Then a certain amount of Ru_3_(CO)_12_ (9.37 mg) dispersed in MeOH solution was added into the above suspension solution dropwise with a ruthenium loading of 1.5 wt%. After magnetic stirring for 12 h at room temperature, rotary evaporation treatment was executed to remove the MeOH solvent. After being dried in a vacuum oven at 60 °C for 24 h, the RCN sample was obtained after a further thermally treatment in H_2_ atmosphere (H_2_ 20%, Ar 80%) at 400 °C for 2 h with a heating rate of 5 °C/min. The obtained product was stored in the glass bottle for further use.

### Synthesis of Ru-RuO_x_ NPs/C_3_N_4_ (RRCN)

The RRCN sample was obtained from RCN sample through a partial oxidation thermally treatment. Typically, the RCN powder (100 mg) was tiled in the porcelain boat. And then, the boat was heated at 200 °C in a muffle furnace for 100 minutes. The obtained product was stored in the glass bottle for further use.

### Characterization methods

Transmission electron microscopy (TEM) analysis was performed on a JEOL-2100F transmission electron microscope at an accelerating voltage of 200 kV. Energy dispersive spectra (EDS-mapping) were performed on a Talos F200X transmission electron microscope at an accelerating voltage of 200 kV. The high-angle annular dark-field scanning transmission electron microscopy (AC-HAADF-STEM) was performed on a JEM-ARM200F instrument (University of Science and Technology if China) with a spherical aberration corrector. X-ray photoelectron spectroscopy (XPS) measurements were carried out on an ESCALAB 250Xi instrument equipped with a Mg Kα source (*hv* = 1253.6 eV). The binding energy scale of all measurements was calibrated by referencing C 1*s* to 284.8 eV. Powder X-ray diffraction (XRD) patterns were recorded on a ESCALAB 250Xi spectrometer with an excitation source of monochromatized Al Ka (*hv* = 1486.6 eV) and a pass energy of 30 eV. The values of binding energies were calibrated with the C 1*s* peak of contaminant carbon at 284.80 eV. Elements quantitative analysis were carried out by an inductively coupled plasma atomic emission spectroscopy (ICP-AES) analysis of PerkinElmer Model Optima 3000DV. UV-Vis diffuse reflectance spectra (DRS) were collected on a Shimadzu DUV-3700 spectrophotometer with BaSO_4_ as the reflectance standard. Photoluminescence (PL) measurements were recorded with an excitation wavelength of 360 nm.

### Transient absorption (TA) measurements and kinetics fitting

Femtosecond TA measurements were based on a regenerative amplified Ti: sapphire laser system (Coherent; 800 nm, 35 fs, 6 mJ/pulse, and 1 kHz repetition rate) and a TA spectrometer (femto-TA100; Time-Tech Spectra LLC). Before TA measurement, we well-dispersed all of the samples in water to form the investigated systems, and quantitatively tuned the absorption of the samples to ~0.5 OD at 350 nm by steady-state absorption spectra. In addition, the pump energy density we adopted in the pump-probe experiments is 336 μJ/cm^2^. Briefly, the 800 nm output pulse from the amplifier was split in two parts. One part was used to pump a TOPAS Optical Parametric Amplifier (OPA) which generated wavelength-tunable pump beams. The other part was further split into two beams. One beam was attenuated with an N.D. filter and was focused onto a 2-mm-thick sapphire window to generate a white light continuum (WLC) as the probe beam. The probe beam was focused with an Al parabolic mirror onto the sample. After the sample, it was collimated and then focused into a fiber-coupled spectrometer with CMOS sensors and detected at a frequency of 1 KHz. The intensity of the pump pulse used in the experiment was controlled by N.D. filters. The delay between the pump and probe pulses was controlled by a motorized delay stage. The pump pulses were chopped by a synchronized chopper at 500 Hz. Samples were placed in 1 mm cuvettes and were vigorously stirred during all the measurements.

For the samples under 350 nm excitation, different exponentials are required to fit the bleach recovery kinetics:

1 $$S(t)\propto \mathop{\sum}\limits_{{{{{{\rm{i}}}}}}}^{{{{{{\rm{j}}}}}}}{{{{{{\rm{A}}}}}}}_{{{{{{\rm{i}}}}}}}\cdot {{{{{{\rm{e}}}}}}}^{-{{{{{{\rm{k}}}}}}}_{{{{{{\rm{i}}}}}}}t}$$

where *A*_i_, *k*_i_ are the amplitude and rate constant of the *i*-th component, respectively.

The decay kinetics of CN are fitted using a mono-exponential decay function:

2 $$S(t)\propto {{{{{\rm{A}}}}}}\cdot {e}^{{-{{{{{\rm{k}}}}}}}_{1}t}$$

Here, k_1_ is the intrinsic decay rate constant;

The decay kinetics of RCN are fitted using a two-exponential decay function

3 $$S(t)\propto {{{{{{\rm{A}}}}}}}_{1}\cdot {{{{{{\rm{e}}}}}}}^{{-{{{{{\rm{k}}}}}}}_{1}t}+{{{{{{\rm{A}}}}}}}_{2}\cdot {{{{{{\rm{e}}}}}}}^{{-{{{{{\rm{k}}}}}}}_{2}t}$$

Here k_2_ are the electron transfer rate constant from CN to Ru.

The decay kinetics of RRCN are fitted using a triple-exponential decay function

4 $$S(t)\propto {{{{{{\rm{A}}}}}}}_{1}\cdot {{{{{{\rm{e}}}}}}}^{{-{{{{{\rm{k}}}}}}}_{1}t}+{{{{{{\rm{A}}}}}}}_{2}\cdot {{{{{{\rm{e}}}}}}}^{{-{{{{{\rm{k}}}}}}}_{2}t}{+{{{{{\rm{A}}}}}}}_{3}\cdot {{{{{{\rm{e}}}}}}}^{{-{{{{{\rm{k}}}}}}}_{3}t}$$

Here k_3_ are the hole transfer rate constant from CN to RuO_x_.

The fitting parameters are tabulated in Table S2; note that the constants in the above equations are all converted to time constants in the table.

### In-situ XAFS measurement

XAFS spectra at the Ru *K*-edge (22117 eV) were measured at the BL14W1 beamline of Shanghai Synchrotron Radiation Facility (SSRF), China. The storage ring of SSRF was operated at 3.5 GeV with a maximum electron current of 250 mA. The hard X-ray was monochromatized with a Si (311) double crystal monochromator. During the XAFS measurements, we seriously calibrate the position of absorption edge (*E*_0_) using Ru foil, and all the XAFS data were collected during one period of beam time.

### XAFS data analysis

The acquired EXAFS data were processed according to the standard procedures using the ATHENA module implemented in the IFEFFIT software packages^44^. The *k*^2^-weighted *χ*(*k*) data in the *k*-space ranging from 3.43 to 12.70 Å^−1^ were Fourier transformed to real (R) space using a hanning windows (d*k* = 1.0 Å^−1^) to separate the EXAFS contributions from different coordination shells. To obtain the detailed structural parameters around Ru atom in the as-prepared samples, quantitative curve-fittings were carried out for the Fourier transformed *k*^2^*χ*(*k*) in the R-space using the ARTEMIS module of IFEFFIT^45^. Effective backscattering amplitudes *F*(*k*) and phase shifts *Φ*(*k*) of all fitting paths were calculated by the ab initio code FEFF8.0^46^. For all the samples, a *k* range of 3.43–12.70 Å^−1^ was used and curve fittings were done in the R-space within the *R* range of 1.3−2.8 Å for *k*^2^-weighted *χ*(*k*) functions. The number of independent points is:

5 $${N}_{{{{{{\rm{ipt}}}}}}}=\frac{2\times \varDelta k\times \varDelta R}{\pi }=\frac{2\times (12.70-3.43)\times (2.8-1.3)}{\pi }=8.86$$

As for the RRCN-*T*_t_ (t = 0, 10, 20, 30, 50, 100 min) samples, the Fourier-transformed curves showed one or two single prominent coordination peaks at ~1.57 Å and 2.42 Å assigned to the Ru-O and Ru-Ru coordination. Two separate Ru-O and Ru-Ru scatting paths were included for fitting. During the curve fitting for the RRCN-*T*_t_ samples, Debye-Waller factors (*σ*^2^), coordination numbers (CN), interatomic distances (*R*), and energy shift ($\triangle$*E*_0_) were treated as adjustable parameters for the Ru-O and Ru-Ru paths. The number of adjustable parameters was *N*_para_ = 4 + 4 = 8, less than the *N*_ipt_.

### Photocatalytic activity measurement

The photocatalytic H_2_ evolution performances of the as-obtained catalysts are evaluated in a closed top-irradiation-type photoreactor (Pyrex glass) connected to a gas circulation system. Typically, 20 mg of the RRCN sample were well-dispersed in 100 mL deionized water without any sacrificial agents after sonication for 1 h, then 300 rpm magnetic stirring was used to ensure the homogeneity of aqueous suspension without sedimentation. As for photocatalytic hydrogen evolution of RCN samples, 15% TEOA in volume was added as the hole sacrifice agent. The reaction solution was continuously in Ar-purged flow to remove air completely, and then irradiated by a 300 W Xe-lamp (PLS-SXE 300, Beijing perfectlight Co. Ltd, China). The generated hydrogen was measured by a gas chromatograph (GC) equipped with a thermal conduction detector (TCD) using Ar as carrier gas.

The quantum efficiency (QE) was calculated from equation as follows^47^,

6 $${{{{{\rm{QE}}}}}}=\frac{2\times {{{{{\rm{the}}}}}}\,{{{{{\rm{number}}}}}}\,{{{{{\rm{of}}}}}}\,{{{{{\rm{evolved}}}}}}\,{{{{{{\rm{H}}}}}}}_{2}\,{{{{{\rm{molecules}}}}}}}{{{{{{\rm{the}}}}}}\,{{{{{\rm{number}}}}}}\,{{{{{\rm{of}}}}}}\,{{{{{\rm{incident}}}}}}\,{{{{{\rm{photons}}}}}}}\times 100\%$$

Several band-pass filters (FWHM = 15 nm) were employed to achieve a different incident light wavelength under a 300 W Xe lamp for measurement of the quantum efficiency. The average intensity of each irradiation wavelength was determined by an optical power meter (PM100D, Thermal Powermeter Head, THORLABS).

For full spectrum measurement, a 300 W xenon arc lamp (PLS-SXE300/300UV) with a standard AM1.5 filter, outputting the light density of 100 mW/cm^2^, was used as illumination source to trigger the photocatalytic reaction for pristine CN, RCN, and RRCN samples. Subsequently, a 400 nm cutoff filter was added to remove the light whose wavelength is shorter than 400 nm, and then the visible-light region of ≥400 nm was obtained as illumination source to activate photocatalytic reaction.

### Photoelectrochemical measurement methods

Electrochemical measurements were performed using an electrochemical workstation (Model CHI 760E, CH Instruments, Inc., Austin, TX) with a stand three-electrode photoelectrochemical cell and was used to record transient photocurrent behavior of the samples, where the prepared electrodes immersed in a sodium sulfate electrolyte solution (0.5 M, pH = 6.8), a platinum mesh and Ag/AgCl (saturated KCl) act as the working, counter, and reference electrode, respectively. The working electrodes were prepared as follows: Approximate 5 mg as-synthesized catalysts were ultrasonically dispersed in 1 mL of 3:1 (volume ratio) DI-water/ethanol mix solvent with 80 μL of Nafion solution (5%), then the mix ink (~150 μL) was uniformly dropped onto a 1 × 2 cm fluorine-doped tin oxide (FTO) substrate as work electrode and then died in an oven at 60 °C. The photo-response of the prepared photoelectrodes (*I*–*t*) was operated by measuring the photocurrent densities under chopped light irradiation (light on/off cycles: 20 s) at a bias potential of 0.3 V vs. Ag/AgCl.

## Supplementary information

Supplementary Information

## Data Availability

The data that support the findings of this study are available within the article and its Supplementary Information. The source data are available from the corresponding authors upon reasonable request.
