# Supplementary material for: In situ constructing atomic interface in ruthenium-based amorphous hybrid-structure towards solar hydrogen evolution
Source: Nat Commun. 2023 Mar 28;14:1720. doi: 10.1038/s41467-023-37451-7 (PMC10050010; doi:10.1038/s41467-023-37451-7)
Supplement: Supplementary file 1 — Supplementary Information [file 41467_2023_37451_MOESM1_ESM.pdf]

Supplementary Information for:

In situ constructing atomic interface in ruthenium-based amorphous hybrid-structure towards solar hydrogen evolution

Dong Liu[1], Tao Ding[1*], Lifeng Wang[2], Huijuan Zhang[1], Li Xu[1], Beibei Pang[1], Xiaokang Liu[1*], Huijuan Wang[3], Junhui Wang[2], Kaifeng Wu[2], and Tao Yao[1*]

[1]National Synchrotron Radiation Laboratory, University of Science and Technology of China, Hefei 230029, PR China

[2]State Key Laboratory of Molecular Reaction Dynamics and Dynamics Research Center for Energy and Environmental Materials, Dalian Institute of Chemical Physics, Chinese Academy of Sciences, Dalian, Liaoning 116023, PR China

[3]Experimental Center of Engineering and Materials Science, University of Science and Technology of China, Hefei 230026, PR China

Email: dingtao@ustc.edu.cn; liuxk@ustc.edu.cn; yaot@ustc.edu.cn

Content list:

1. Supplementary Methods

2. Supplementary Figures

3. Supplementary Tables

4. References

1. Supplementary Methods

Chemicals. All the chemicals were used without further purification. Urea (CH_4N_2O), methanol (CH_3OH), triethanolamine (TEOA), were acquired from Sinopharm Chemical Reagent Co., Ltd. Ruthenium Carbonyl ($Ru_3(CO)_{12}$), ruthenium (III) chloride ($RuCl_3$), were acquired from Sigma-Aldrich.

Synthesis of g-C_3N_4 (CN). The carbon nitride (g-C_3N_4) was first synthesized as a support. Briefly, a certain amount of urea was put into a covered crucible and then heated to 600 °C for 2 h in a muffle furnace with a heating rate of 5 °C/min. After cooling to room temperature, the obtained flavescent powder was washed with deionized water to eliminate the dust static electricity and remove impurities, whereafter, collected by filtration and finally dried under vacuum at 60 °C.

Synthesis of Ru NPs/C_3N_4 (RCN). In a typical synthesis, the g-C_3N_4 powder (300 mg) was dispersed in 150 ml MeOH and sonicated for 12 h. Then a certain amount of $Ru_3(CO)_{12}$ (9.37 mg) dispersed in MeOH solution was added into the above suspension solution dropwise with a ruthenium loading of 1.5 wt%. After magnetic stirring for 12 h at room temperature, rotary evaporation treatment was executed to remove the MeOH solvent. After being dried in a vacuum oven at 60 °C for 24 h, the RCN sample was obtained after a further thermally treatment in H_2 atmosphere (H_2 20%, Ar 80%) at 400 °C for 2 h with a heating rate of 5 °C/min. The obtained product was stored in the glass bottle for further use.

Synthesis of Ru-RuO_x NPs/C_3N_4 (RRCN). The RRCN sample was obtained from RCN sample through a partial oxidation thermally treatment. Typically, the RCN powder (100 mg) was tiled in the porcelain boat. And then, the boat was heated at 200 °C in a muffle furnace for 100 minutes. The obtained product was stored in the glass bottle for further use.

Characterization methods. Transmission electron microscopy (TEM) analysis was performed on a JEOL-2100F transmission electron microscope at an accelerating voltage of 200 kV. Energy dispersive spectra (EDS-mapping) was performed on a Talos F200X

transmission electron microscope at an accelerating voltage of 200 kV. The high-angle annular dark-field scanning transmission electron microscopy (AC-HAADF-STEM) was performed on a JEM-ARM200F instrument (University of science and Technology if China) with a spherical aberration corrector. X-ray photoelectron spectroscopy (XPS) measurements was carried out on an ESCALAB 250Xi instrument equipped with a Mg Kα source (hv = 1253.6 eV). The binding energy scale of all measurements was calibrated by referencing C 1*s* to 284.8 eV. Powder X-ray diffraction (XRD) patterns were recorded on a ESCALAB 250Xi spectrometer with an excitation source of monochromatized Al Ka (hv = 1486.6 eV) and a pass energy of 30 eV. The values of binding energies were calibrated with the C 1*s* peak of contaminant carbon at 284.80 eV. Elements quantitative analysis were carried out by an inductively coupled plasma atomic emission spectroscopy (ICP-AES) analysis of PerkinElmer Model Optima 3000DV. UV-Vis diffuse reflectance spectra (DRS) were collected on a Shimadzu DUV-3700 spectrophotometer with $BaSO_4$ as the reflectance standard. Photoluminescence (PL) measurements were recorded with an excitation wavelength of 360 nm.

Transient absorption (TA) measurements and kinetics fitting. Femtosecond TA measurements were based on a regenerative amplified Ti: sapphire laser system (Coherent; 800 nm, 35 fs, 6 mJ/pulse, and 1 kHz repetition rate) and a TA spectrometer (femto-TA100; Time-Tech Spectra LLC). Before TA measurement, we well dispersed all of the samples in water to form the investigated systems, and quantitatively tuned the absorption of the samples to ~0.5 OD at 350 nm by steady-state absorption spectra. In addition, the pump energy density we adopted in the pump-probe experiments is 336 $\mu J/cm^2$. Briefly, the 800 nm output pulse from the amplifier was split in two parts. One part was used to pump a TOPAS Optical Parametric Amplifier (OPA) which generated wavelength-tunable pump beams. The other part was further split into two beams. One beam was attenuated with an N.D. filter and was focused onto a 2-mm-thick sapphire window to generate a white light continuum (WLC) as the probe beam. The probe beam was focused with an Al parabolic

mirror onto the sample. After the sample, it was collimated and then focused into a fiber-coupled spectrometer with CMOS sensors and detected at a frequency of 1 KHz. The intensity of the pump pulse used in the experiment was controlled by N.D. filters. The delay between the pump and probe pulses was controlled by a motorized delay stage. The pump pulses were chopped by a synchronized chopper at 500 Hz. Samples were placed in 1 mm cuvettes and were vigorously stirred during all the measurements.

For the samples under 350 nm excitation, different exponentials are required to fit the bleach recovery kinetics:

$$S(t) \propto \sum_{i}^{j} A_i \cdot e^{-k_i t} \tag{1}$$

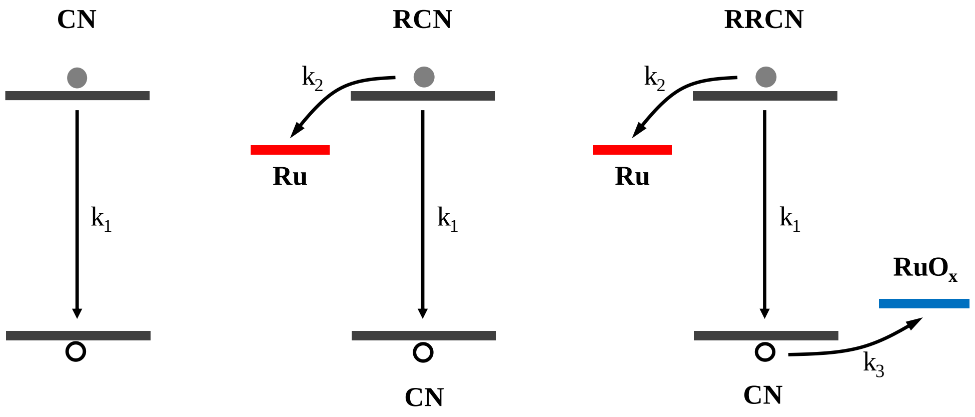

where A_i, k_i are the amplitude and rate constant of the i-th component, respectively.

The decay kinetics of CN are fitted using a mono-exponential decay function:

$$S(t) \propto A \cdot e^{-k_1 t} \tag{2}$$

Here, k_1 is the intrinsic decay rate constant;

The decay kinetics of RCN are fitted using a two-exponential decay function

$$S(t) \propto A_1 \cdot e^{-k_1 t} + A_2 \cdot e^{-k_2 t} \tag{3}$$

Here k_2 are the electron transfer rate constant from CN to Ru.

The decay kinetics of RRCN are fitted using a triple-exponential decay function

$$S(t) \propto A_1 \cdot e^{-k_1 t} + A_2 \cdot e^{-k_2 t} + A_3 \cdot e^{-k_3 t} \tag{4}$$

Here k_3 are the hole transfer rate constant from CN to RuO_x.

The fitting parameters are tabulated in Table S2; note that the constants in the above equations are all converted to time constants in the table.

In-situ XAFS measurement. XAFS spectra at the Ru *K*-edge (22117 eV) were measure at the BL14W1 beamline of Shanghai Synchrotron Radiation Facility (SSRF), China. The storage ring of SSRF was operated at 3.5 GeV with a maximum electron current of 250 mA. The hard X-ray was monochromatized with a Si (311) double crystal monochromator. During the XAFS measurements, we seriously calibrate the position of absorption edge (E_0) using Ru foil, and all the XAFS data were collected during one period of beam time.

XAFS data analysis. The acquired EXAFS data were processed according to the standard procedures using the ATHENA module implemented in the IFEFFIT software packages[1]. The k^2-weighted $\chi(k)$ data in the k-space ranging from 3.43 to 12.70 Å^{-1} were Fourier transformed to real (R) space using a hanning windows (dk = 1.0 Å^{-1}) to separate the EXAFS contributions from different coordination shells. To obtain the detailed structural parameters around Ru atom in the as-prepared samples, quantitative curve-fittings were carried out for the Fourier transformed $k^2\chi(k)$ in the R-space using the ARTEMIS module of IFEFFIT[2]. Effective backscattering amplitudes $F(k)$ and phase shifts $\Phi(k)$ of all fitting paths were calculated by the ab initio code FEFF8.0[3]. For all the samples, a k range of 3.43 – 12.70 Å^{-1} was used and curve fittings were done in the R-space within the R range of 1.3 - 2.8 Å for k^2-weighted $\chi(k)$ functions. The number of independent points is:

$$N_{\text{ipt}} = \frac{2\times\Delta k\times\Delta R}{\pi} = \frac{2\times(12.70\text{-}3.43)\times(2.8\text{-}1.3)}{\pi}=8.86 \quad (5)$$

As for the RRCN-T_t (t = 0, 10, 20, 30, 50, 100 min) samples, the Fourier-transformed curves showed one or two single prominent coordination peaks at ~1.57 Å and 2.42 Å assigned to the Ru-O and Ru-Ru coordination. Two separate Ru-O and Ru-Ru scatting paths were included for fitting. During the curve fitting for the RRCN-T_t samples, Debye-Waller factors (σ^2), coordination numbers (CN), interatomic distances (R) and energy shift (ΔE_0) were treated as adjustable parameters for the Ru-O and Ru-Ru paths. The number of

adjustable parameters was $N_{para} = 4 + 4 = 8$, less than the N_{ipt}.

Photocatalytic activity measurement. The photocatalytic H_2 evolution performances of the as-obtained catalysts are evaluated in a closed top-irradiation-type photoreactor (Pyrex glass) connected to a gas circulation system. Typically, 20 mg of the RRCN sample were well-dispersed in 100 mL deionized water without any sacrificial agents after sonication for 1 h, then 300 rpm magnetic stirring was used to ensure the homogeneity of aqueous suspension without sedimentation. As for photocatalytic hydrogen evolution of RCN samples, 15% TEOA in volume was added as the hole sacrifice agent. The reaction solution was continuously in Ar-purged flow to remove air completely, and then irradiated by a 300 W Xe-lamp (PLS-SXE 300, Beijing perfectlight Co. Ltd, China). The generated hydrogen was measured by a gas chromatograph (GC) equipped with a thermal conduction detector (TCD) using Ar as carrier gas.

The quantum efficiency (QE) was calculated from equation as follows[4],

$$QE = \frac{2\times\text{the number of evolved } H_2 \text{ molecules}}{\text{the number of incident photons}}\times 100\% \qquad (6)$$

Several band-pass filters (FWHM = 15 nm) were employed to achieve a different incident light wavelength under a 300 W Xe lamp for measurement of the quantum efficiency. The average intensity of each irradiation wavelength was determined by an optical power meter (PM100D, Thermal Powermeter Head, THORLABS).

For full spectrum measurement, a 300 W xenon arc lamp (PLS-SXE300/300UV) with a standard AM1.5 filter, outputting the light density of 100 mW/cm^2, was used as illumination source to trigger the photocatalytic reaction for pristine CN, RCN and RRCN samples. Subsequently, a 400 nm cutoff filter was added to remove the light whose wavelength is shorter than 400 nm, and then the visible-light region of $\geq$ 400 nm was obtained as illumination source to activate photocatalytic reaction.

Photoelectrochemical measurement methods. Electrochemical measurements were performed using an electrochemical workstation (Model CHI 760E, CH instruments, Inc.,

Austin, TX) with a stand three-electrode photoelectrochemical cell and was used to record transient photocurrent behavior of the samples, where the prepared electrodes immersed in a sodium sulfate electrolyte solution (0.5 M, pH = 6.8), a platinum mesh and Ag/AgCl (saturated KCl) act as the working, counter and reference electrode, respectively. The working electrodes were prepared as follows: Approximate 5 mg as-synthesized catalysts were ultrasonically dispersed in 1 mL of 3:1 (volume ratio) DI-water/ethanol mix solvent with 80 μL of Nafion solution (5%), then the mix ink (~150 μL) was uniformly dropped onto a 1×2 cm fluorine-doped tin oxide (FTO) substrate as work electrode and then died in an oven at 60 °C. The photo-response of the prepared photoelectrodes (I-t) was operated by measuring the photocurrent densities under chopped light irradiation (light on/off cycles: 20 s) at a bias potential of 0.3 V vs. Ag/AgCl.

2. Supplementary Figures

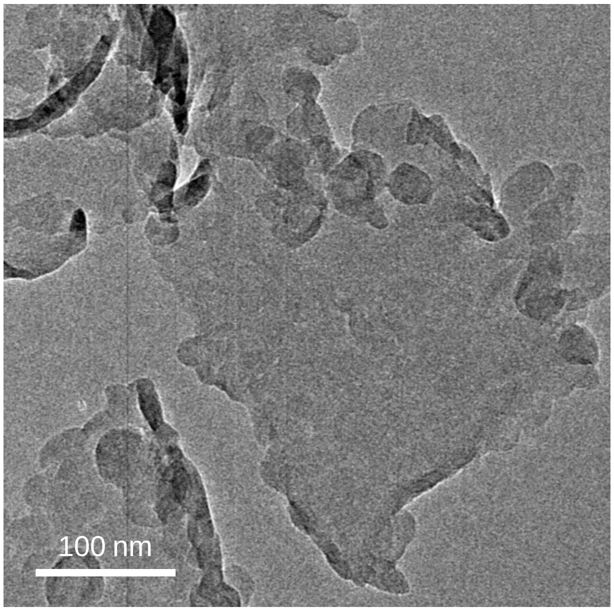

Supplementary Figure 1. Microstructure of the CN support. TEM images of the g-C_3N_4 nanosheet.

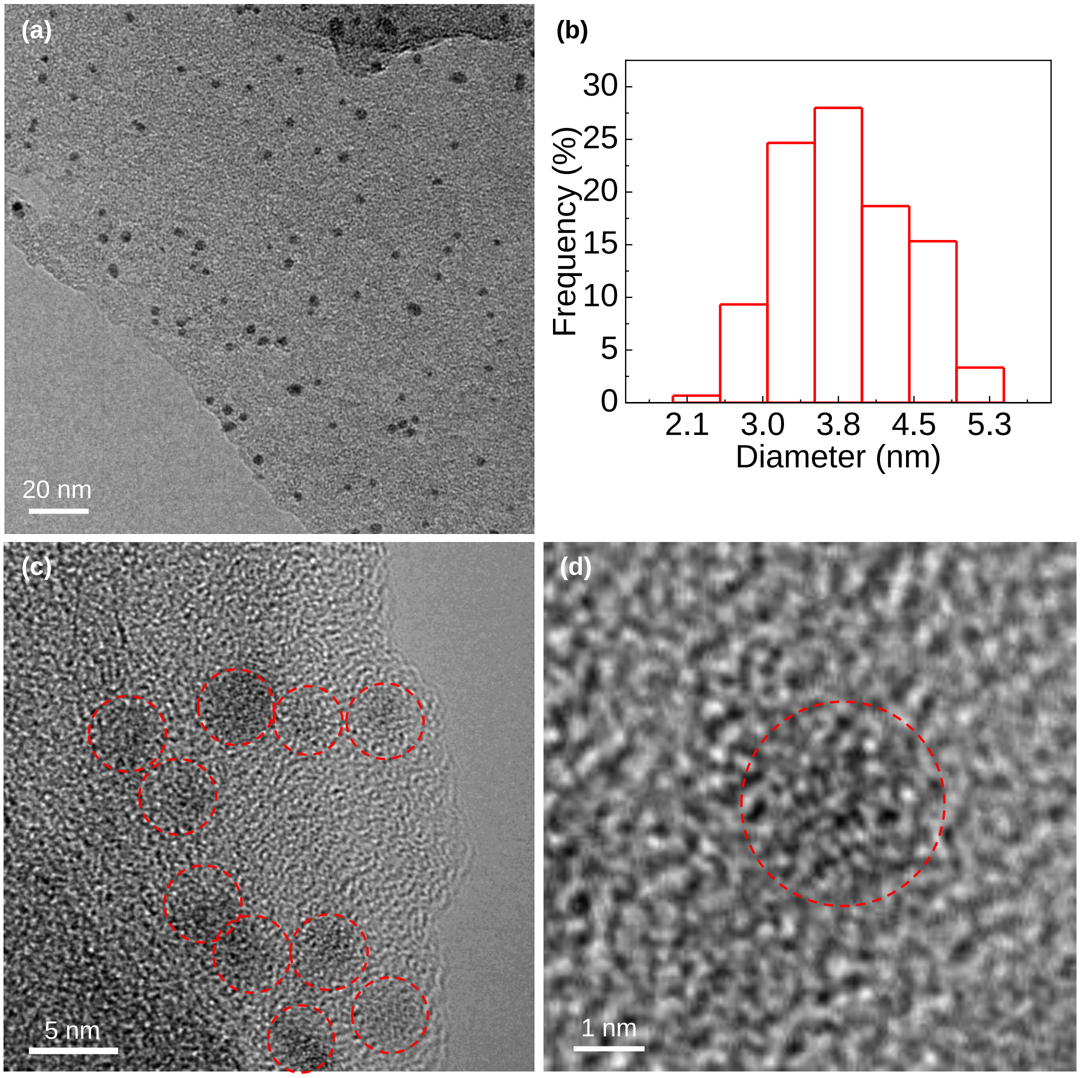

Supplementary Figure 2. Microstructure of the RCN photocatalyst. (a) TEM and (c), (d) HRTEM images of the RCN sample and the corresponding size distribution (b).

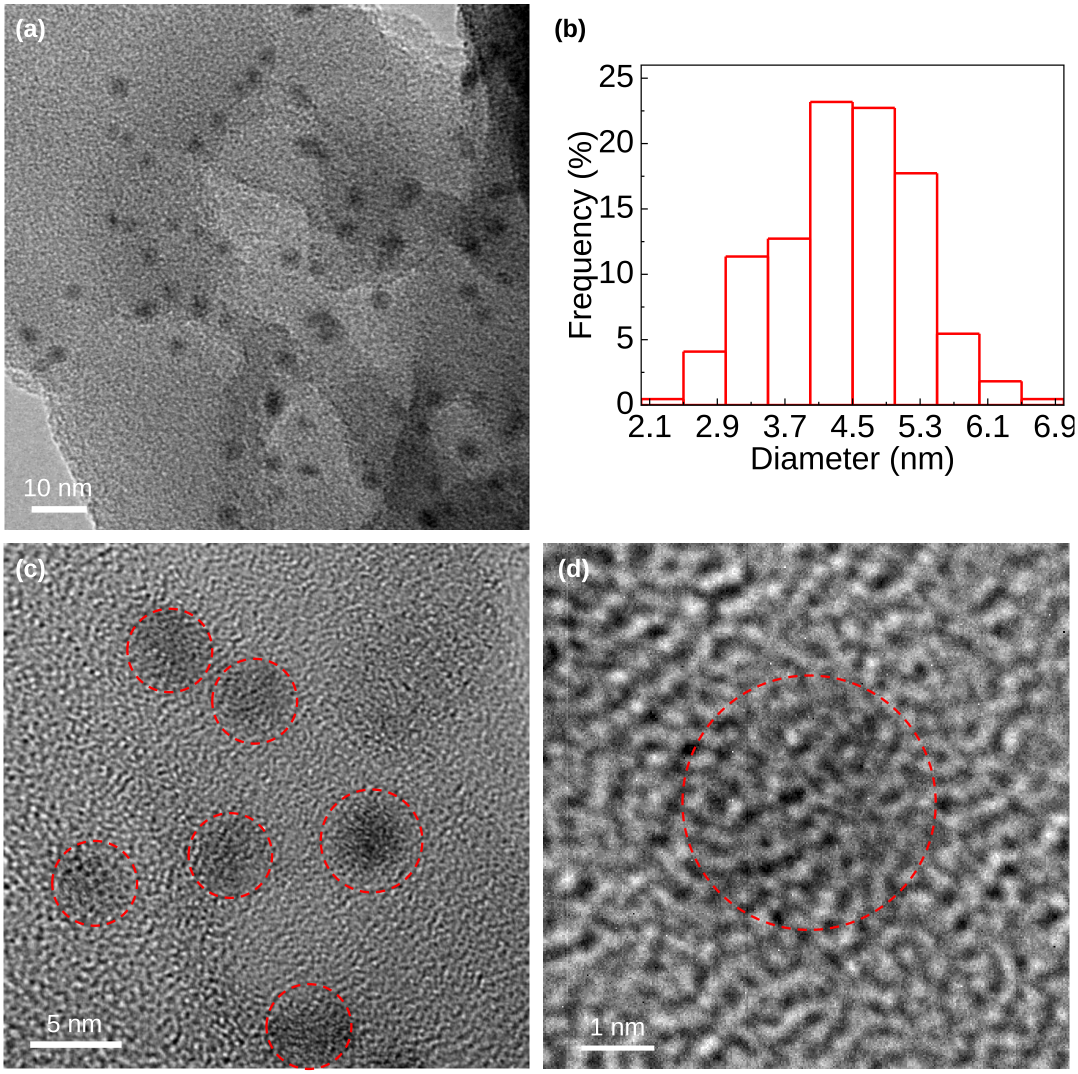

Supplementary Figure 3. Microstructure of the RRCN photocatalyst. (a) TEM and (c), (d) HRTEM images of the RRCN sample and the corresponding size distribution (b).

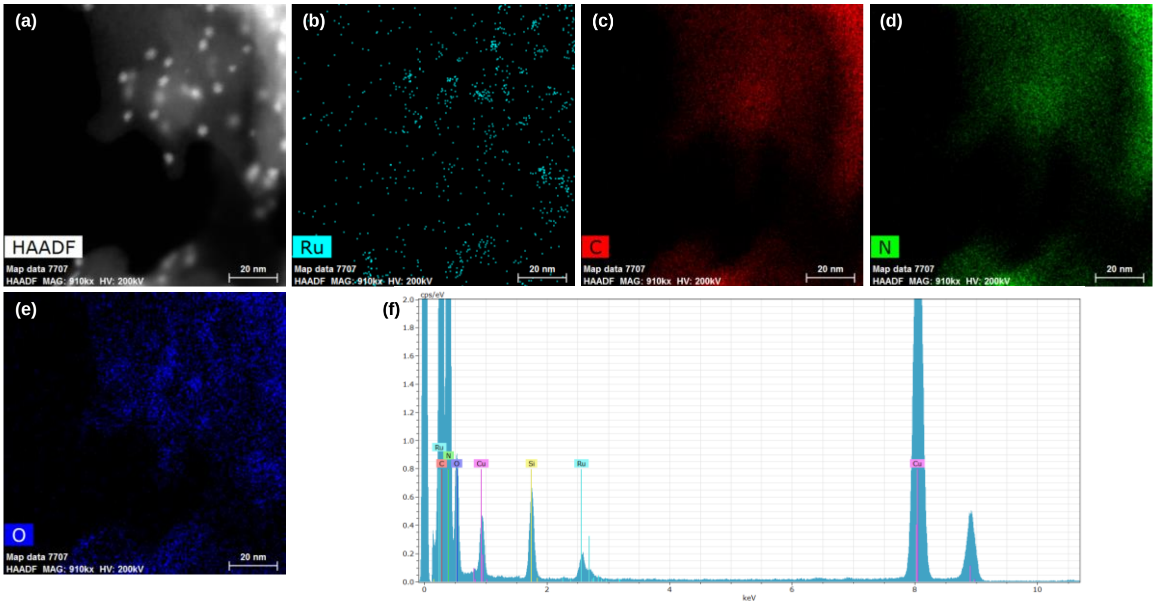

Supplementary Figure 4. Elemental analysis of the RRCN photocatalyst. (a), (b), (c), (d), (e) EDS mapping and corresponding spectrum (f) of RRCN sample.

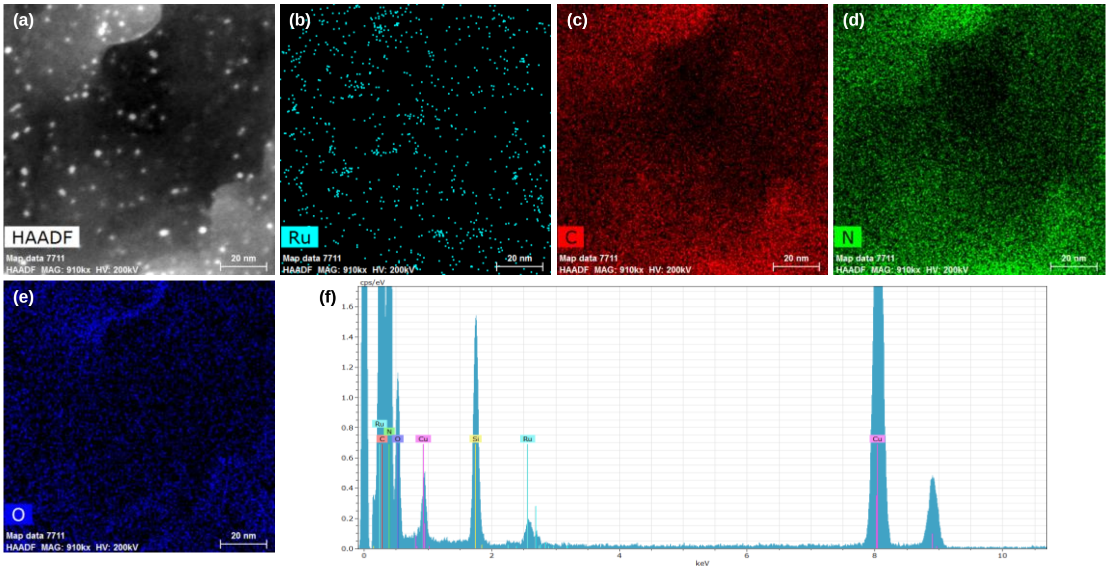

Supplementary Figure 5. Elemental analysis of the RCN photocatalyst. (a), (b), (c), (d), (e) EDS mapping and corresponding spectrum (f) of the RCN sample.

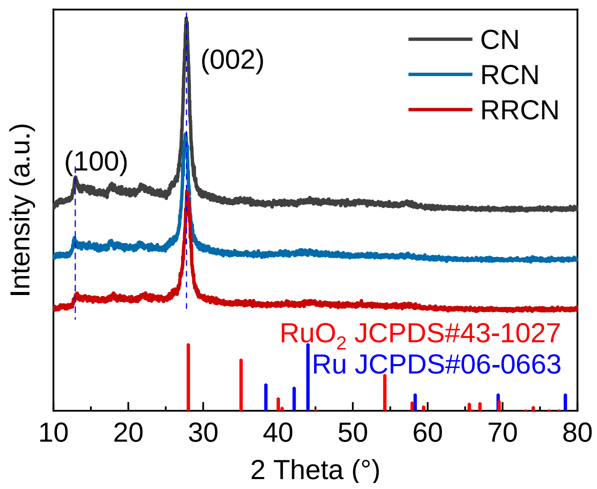

Supplementary Figure 6. Structural characterization of the catalysts. XRD patterns of g-C_3N_4, RCN and RRCN.

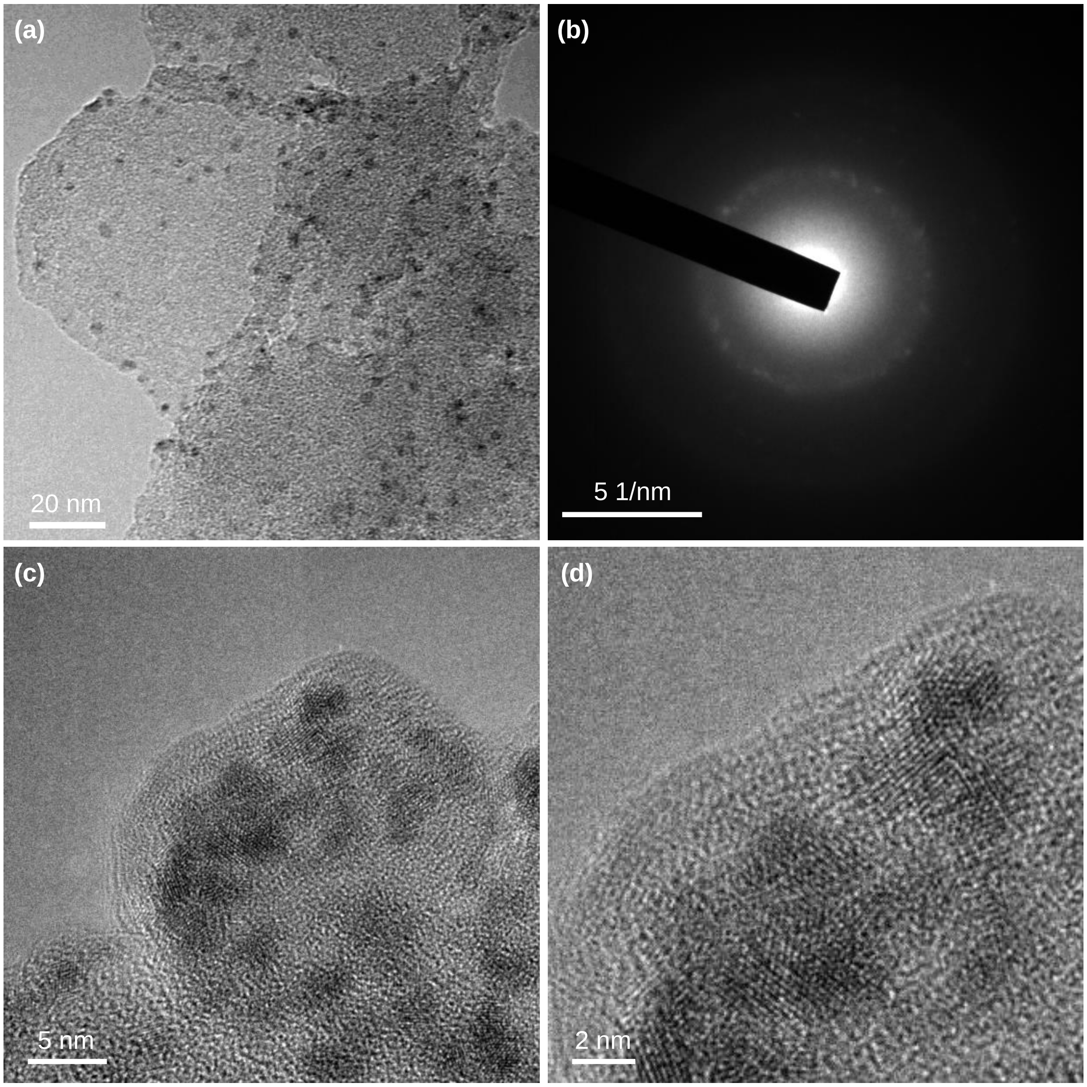

Supplementary Figure 7. Microstructure of the RCN-cry photocatalyst. (a) TEM, (c), (d) HRTEM and (b) SAED images of the RCN-cry sample.

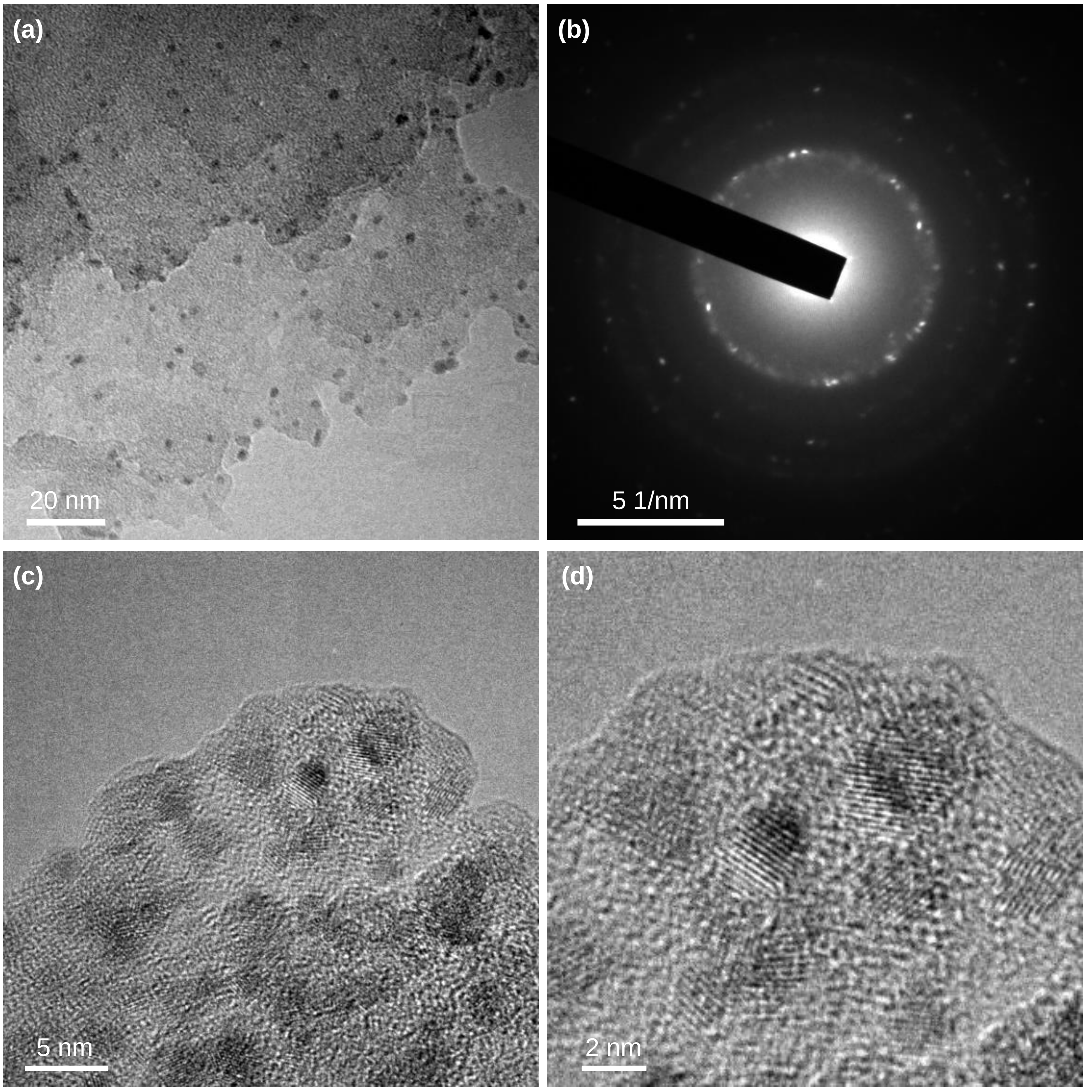

Supplementary Figure 8. Microstructure of the RRCN-cry photocatalyst. (a) TEM, (c), (d) HRTEM and (b) SAED images of the RRCN-cry sample.

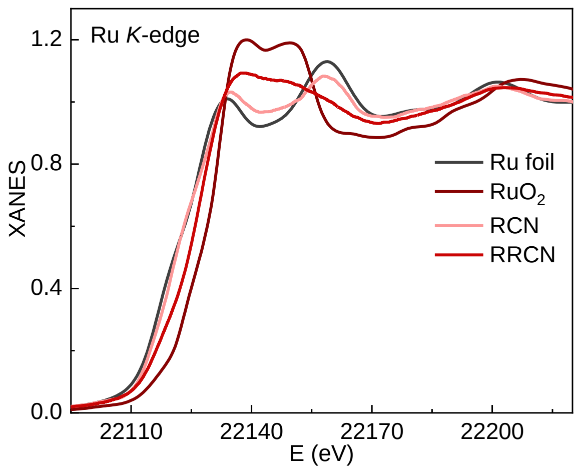

Supplementary Figure 9. XANES characterization of the catalysts. Ru *K*-edge XANES spectra of RCN, RRCN and the referenced standards of Ru foil and RuO_2.

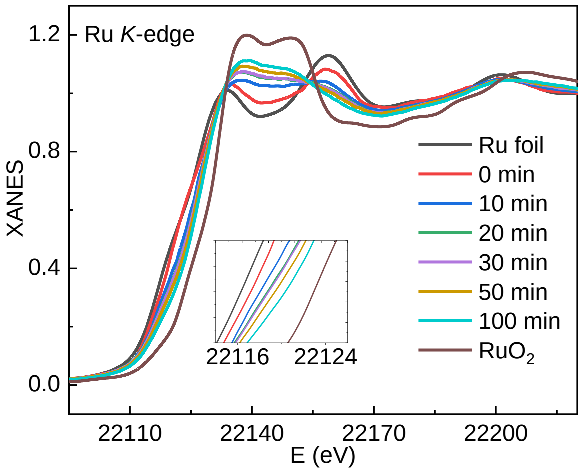

Supplementary Figure 10. In-situ XANES characterization of the catalyst. In-situ XANES spectra recorded at the Ru *K*-edge of RRCN at different oxidation time during the heat process, and the XANES data of the referenced standards of Ru foil and RuO_2.

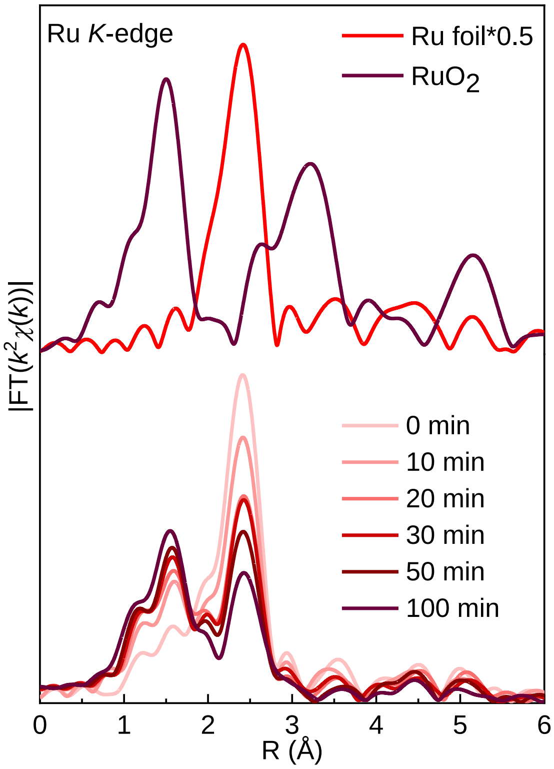

Supplementary Figure 11. In-situ EXAFS characterization of the RRCN catalyst. k^2-weighted Fourier transform (FT) Ru *K*-edge in-situ XANES spectra recorded at the Ru *K*-edge of RRCN at different oxidation time during the heat process (no phase correction).

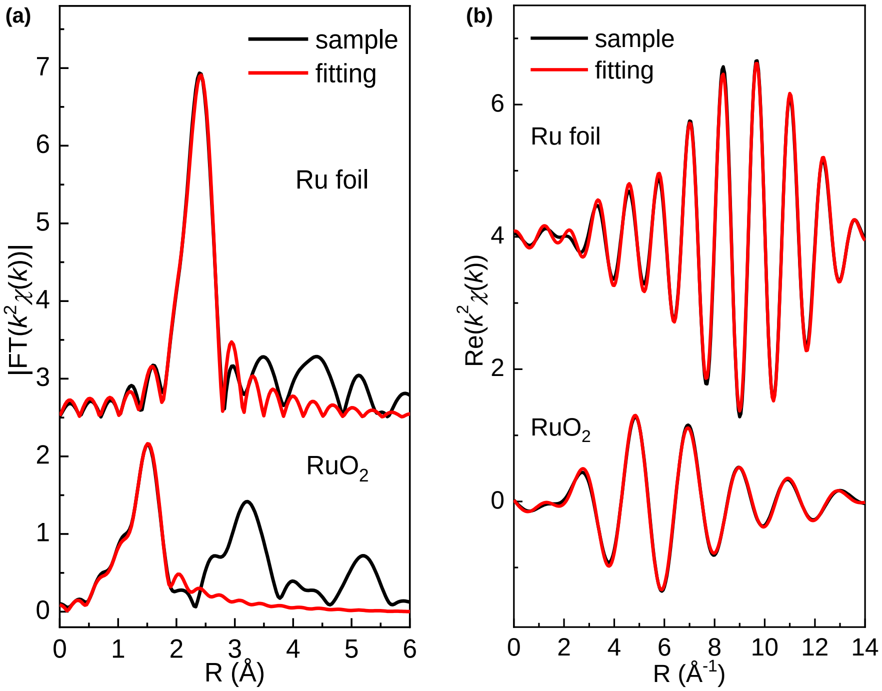

Supplementary Figure 12. EXAFS and fitting results of the Ru standards. (a) Least-squares curve-fitting analysis of EXAFS spectra of Ru foil and RuO_2 at the Ru *K*-edge. (b) Corresponding Re($k^2\chi(k)$) oscillations.

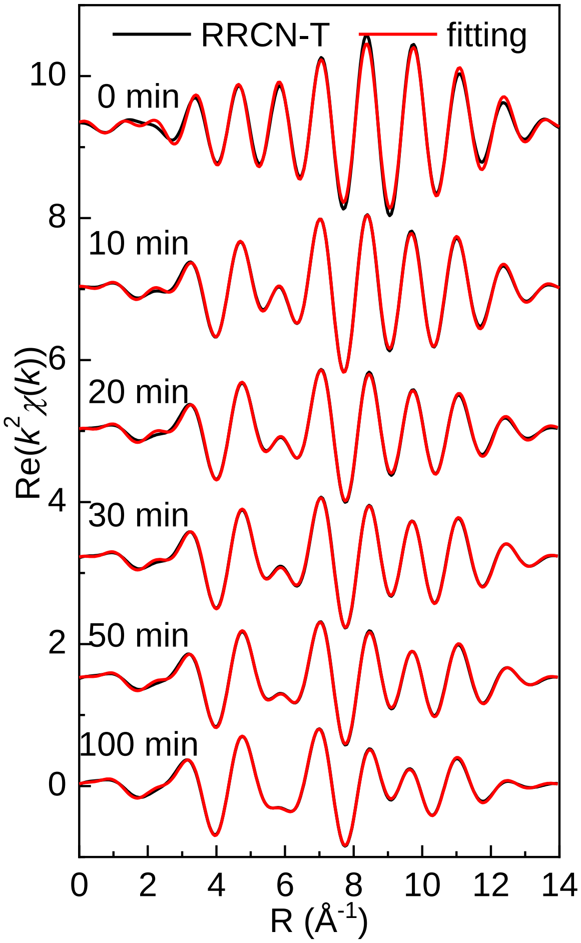

Supplementary Figure 13. EXAFS and fitting results of the RRCN catalyst. Re($k^2\chi(k)$) oscillations of Ru *K*-edge in-situ EXAFS analysis of the RRCN sample.

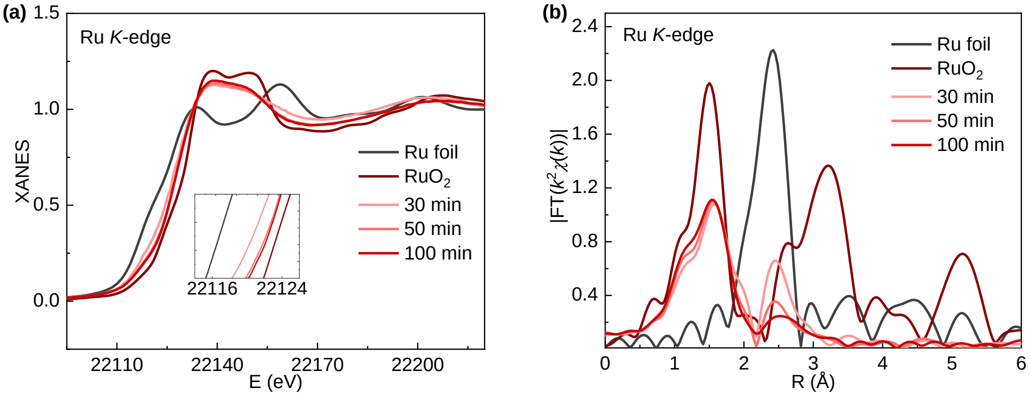

Supplementary Figure 14. In-situ XAFS characterization of the RRCN-cry catalyst. (a) In-situ XANES spectra recorded at the Ru *K*-edge of RRCN-cry at different oxidation time during the heat process, and the XANES data of the referenced standards of Ru foil and RuO_2. Inset: Magnified absorption edge of XANES region. (b) k^2-weighted Fourier transform (FT) Ru *K*-edge in-situ EXAFS spectra of RRCN-cry at different oxidation time during the heat process (no phase correction).

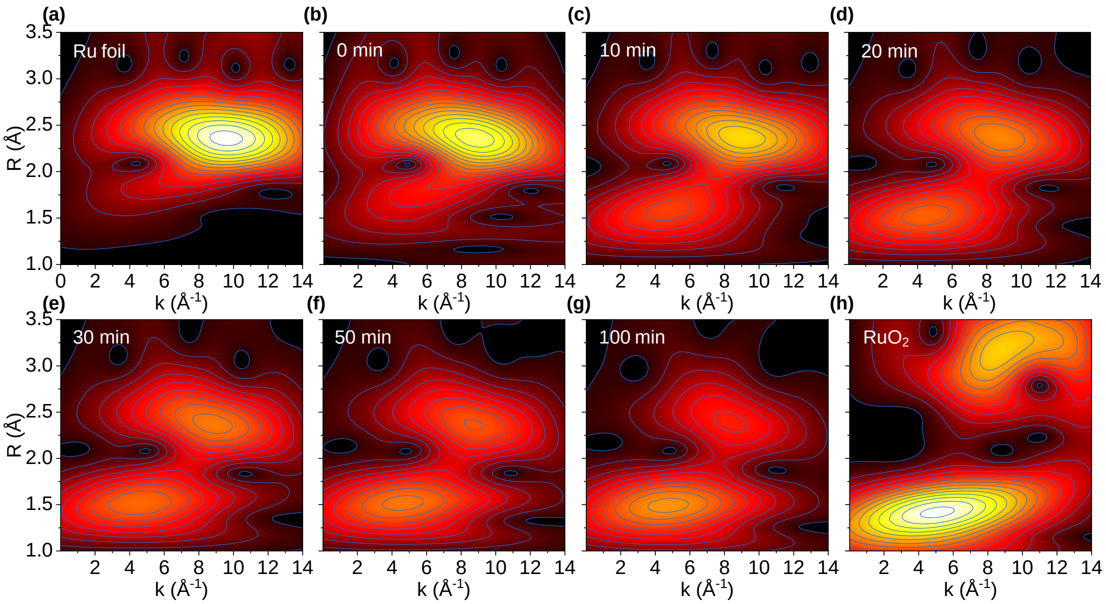

Supplementary Figure 15. Wavelet analysis of the RRCN catalyst. Wavelet analysis of the RRCN sample during in-situ EXAFS states and referenced standards of Ru foil and RuO_2.

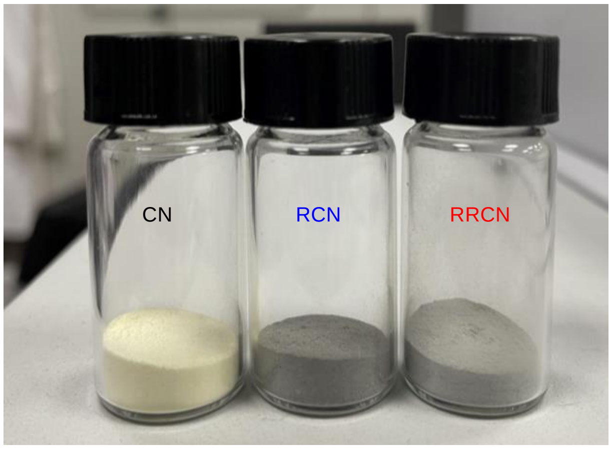

Supplementary Figure 16. Picture of the catalysts. The picture of CN, RCN and RRCN samples.

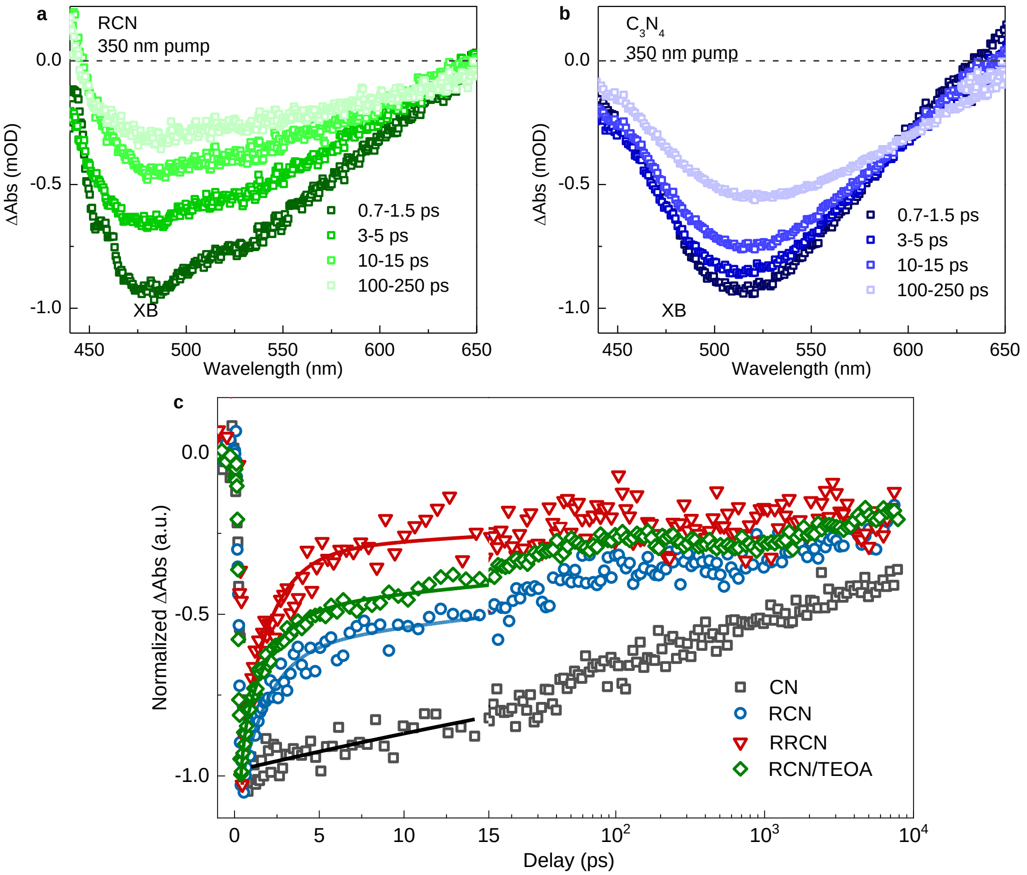

Supplementary Figure 17. TA analysis of the catalysts. (a, b) TA spectra of free C_3N_4, RCN, RRCN and RRCN/TEOA sample measured with 350 nm excitation. (c) TA kinetics probed at the XB of all samples. Before TA measurement, we well dispersed the samples in water to form the investigated systems, and quantitatively tuned the absorption of the samples to ~0.5 OD at 350 nm by steady-state absorption spectra. In addition, the pump energy density we adopted in the pump-probe experiments is 336 μJ/cm^2.

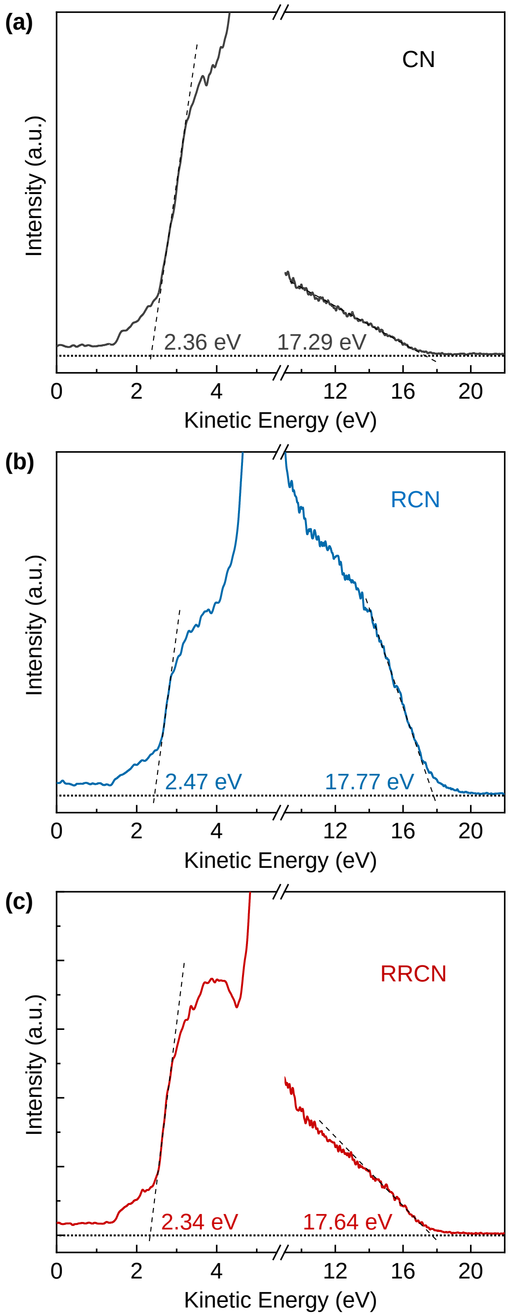

Supplementary Figure 18. Energy band analysis of the catalysts. Low and high kinetic energy region of UPS spectra from (a) CN, (b) RCN and (c) RRCN samples.

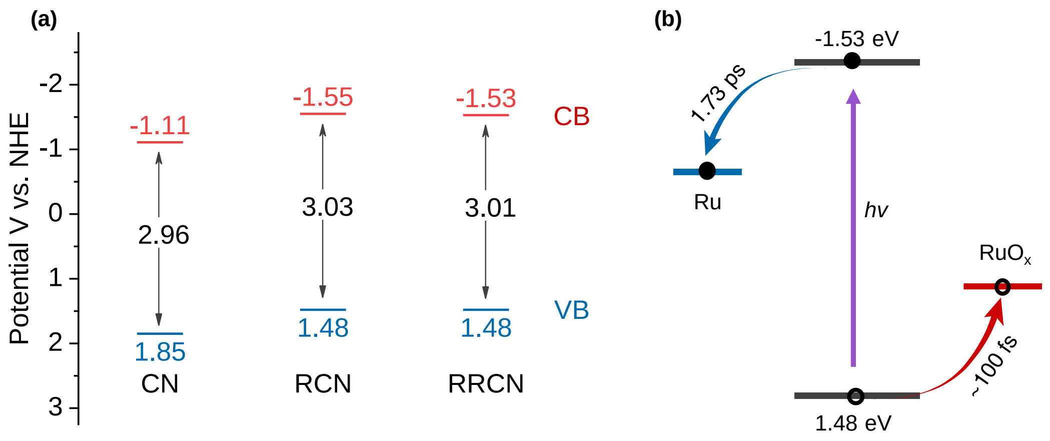

Supplementary Figure 19. Energy band structure of the catalysts. (a) Energy band structure of CN, RCN and RRCN samples. (b) Physical image of charge transfer and energy band structure of RRCN sample.

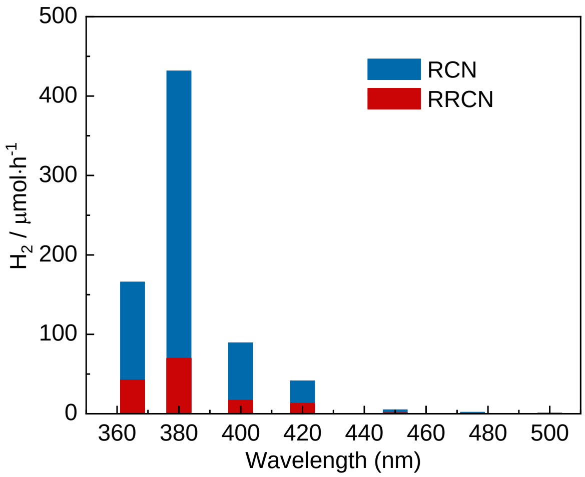

Supplementary Figure 20. Catalytic performance of the catalysts. Hydrogen evolution rates of RCN and RRCN under irradiation with different wavelengths.

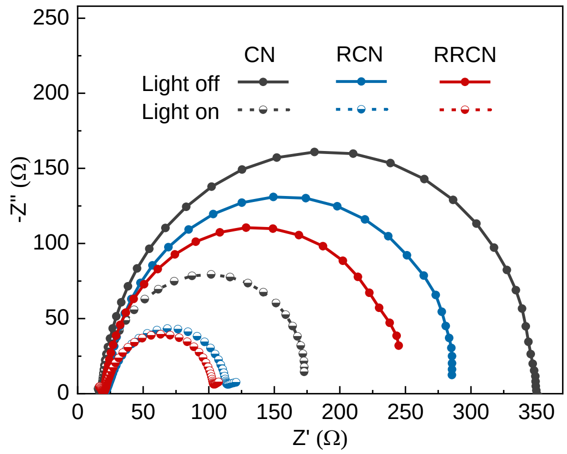

Supplementary Figure 21. Photoelectrochemical performance of the catalysts. Hydrogen evolution rates of RCN and RRCN under irradiation with different wavelengths.

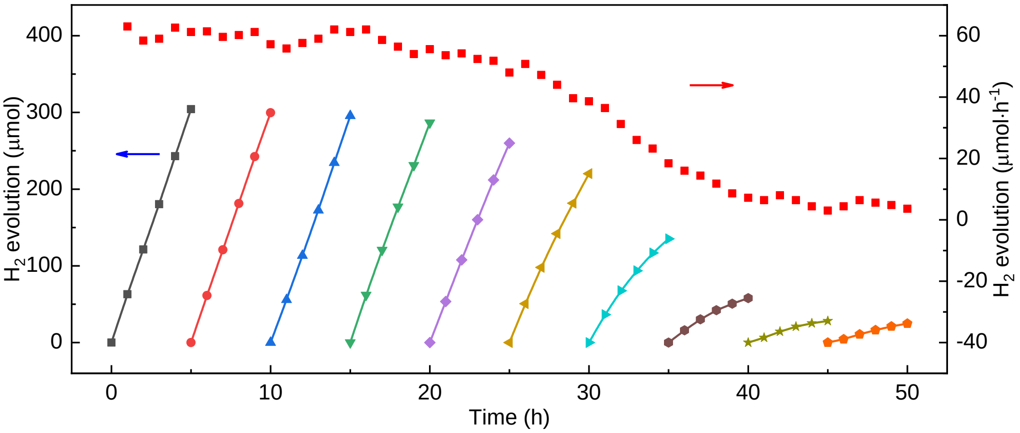

Supplementary Figure 22. Catalytic performance of the catalysts. Long-term recycling performance of the RRCN sample without TEOA.

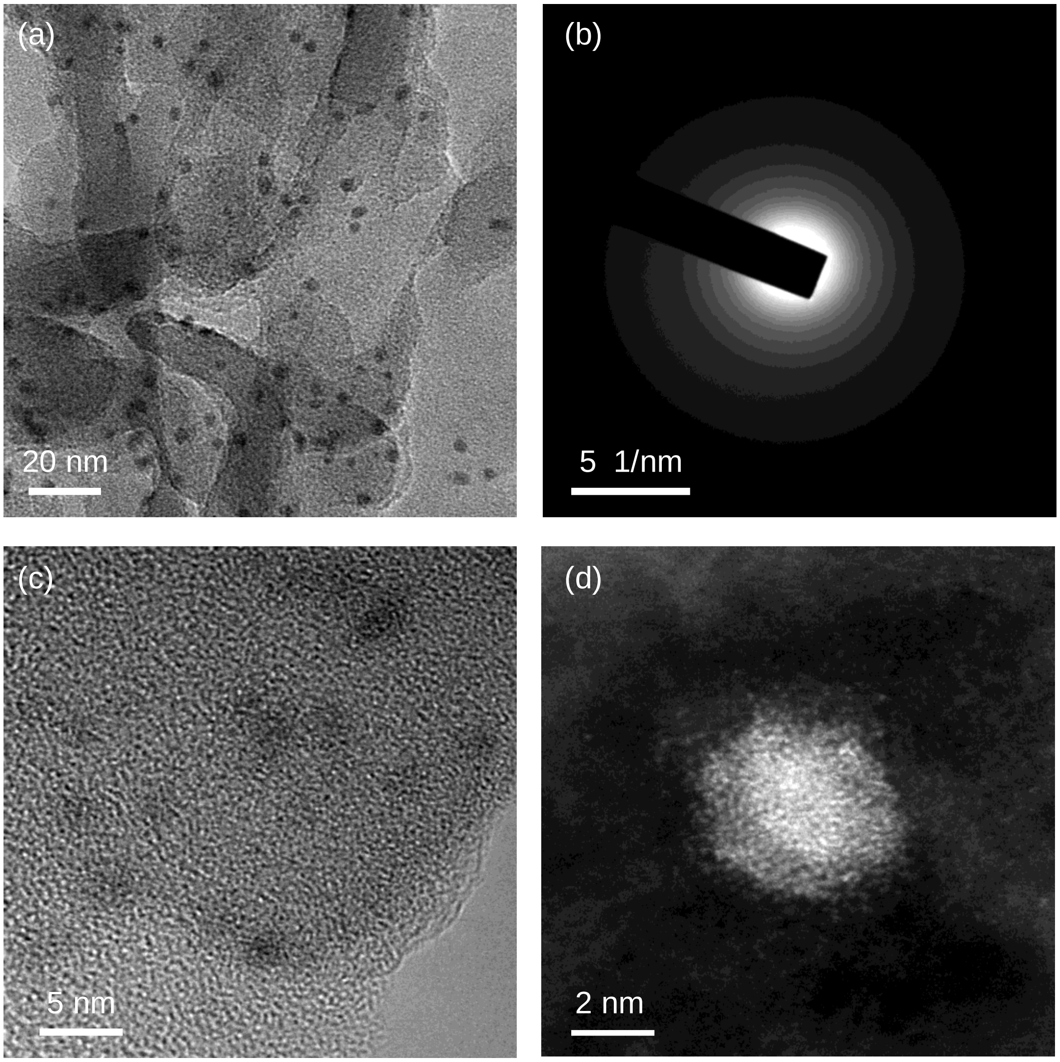

Supplementary Figure 23. Microstructure of the RRCN photocatalyst after reaction. (a) TEM, (b) SAED, (c) HRTEM and (d) AC-HAADF-STEM images of RRCN catalyst after photocatalytic H_2 evolution reaction of 50 h.

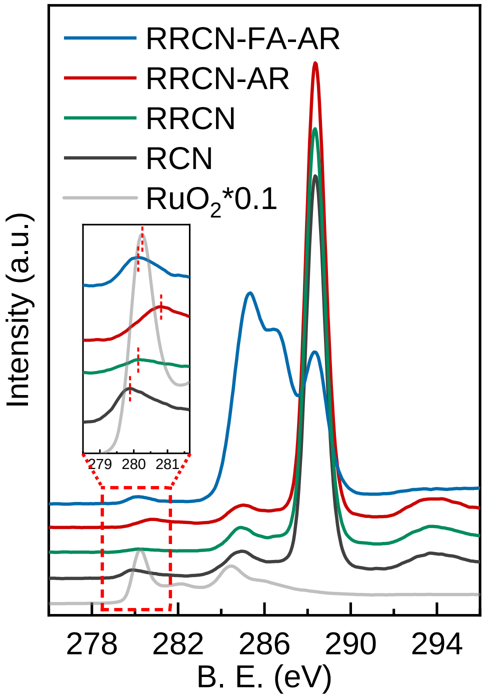

Supplementary Figure 24. Valence state analysis of the catalysts after reaction. The XPS spectra of Ru 3*d* for RuO_2, RCN, RRCN, RRCN-AR (after reaction, 50 h) and RRCN-FA-AR samples (hydrogen evolution reaction coupled with furfuryl alcohol oxidation, after reaction, 50 h).

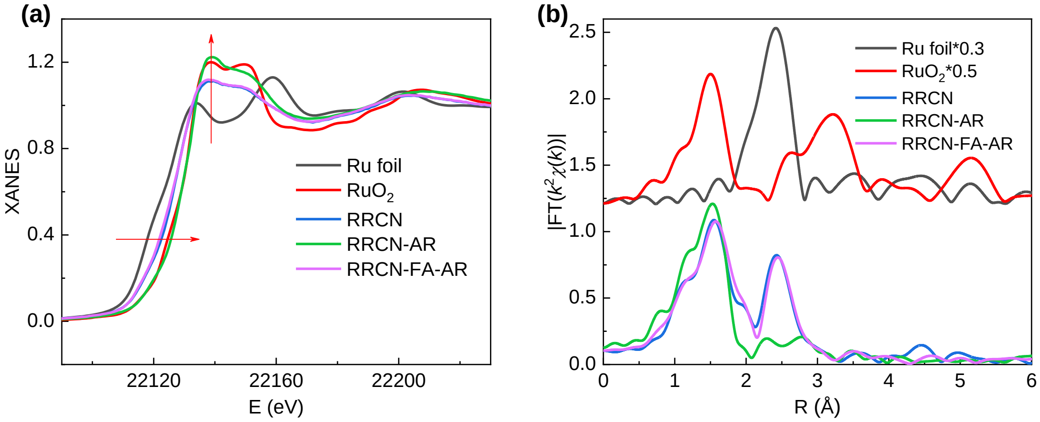

Supplementary Figure 25. XAFS analysis of the catalysts after reaction. The Ru *K*-edge XANES spectra and corresponding k^2-weighted Fourier transforms (FT) spectra for Ru foil, RuO_2, RRCN, RRCN-AR (after reaction, 50 h) and RRCN-FA-AR samples (hydrogen evolution reaction coupled with furfuryl alcohol oxidation, after reaction, 50 h).

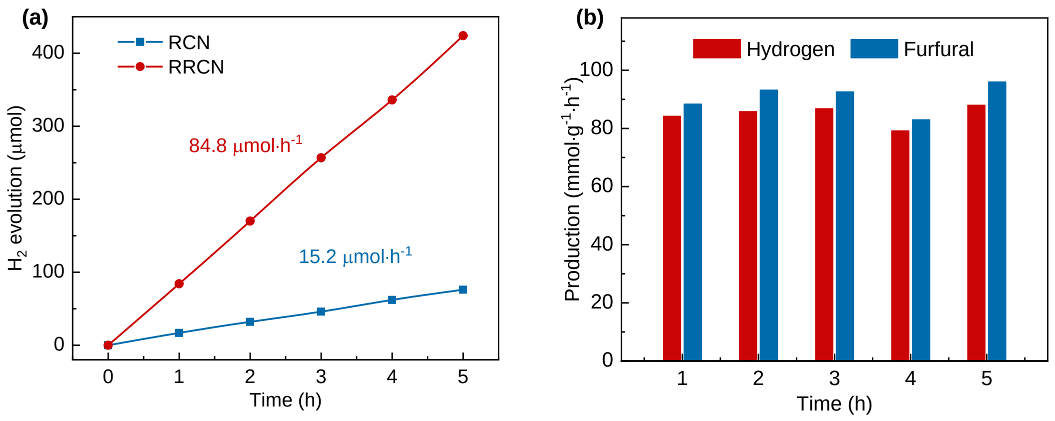

Supplementary Figure 26. Catalytic performance of the catalysts. (a) Typical time course of H_2 productions for RCN and RRCN samples coupled with furfuryl alcohol oxidation. (b) H_2 productions and the furfuryl alcohol oxidation to furfural performance for RRCN samples.

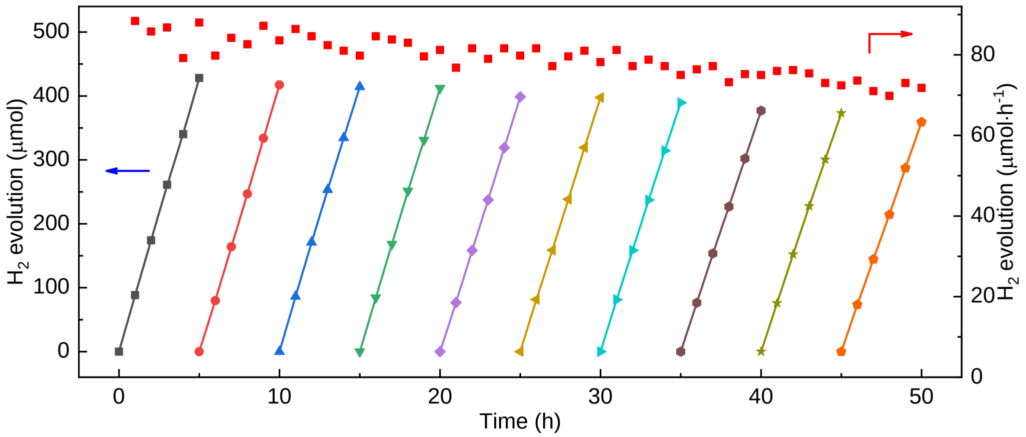

Supplementary Figure 27. Catalytic performance of the catalysts. Long-term recycling performance of RRCN sample in the reaction of H_2 productions coupled with the furfuryl alcohol oxidation to furfural.

3. Supplementary Tables

Supplementary Table 1. Structural parameters of RCN and RRCN at the Ru *K*-edge extracted from quantitative EXAFS curve-fitting using the ARTEMIS module of IFEFFIT. For comparison, the interatomic distance and coordination numbers for the references (Ru foil and RuO_2) calculated their crystallographic structures are also listed.

Samples	Path	CN	*R* (Å)	σ^2 (10^{-3}Å^2)	ΔE_0 (eV)
Ru foil	Ru-Ru	12	2.67	3.3	1.7
RuO_2	Ru-O	6	1.97	3.4	3.2
RCN	Ru-Ru	6.6	2.67	6.5	3.0
RRCN-10	Ru-O	1.7	2.04	6.0	9.5
	Ru-Ru	5.2	2.67	7.8	3.7
RRCN-20	Ru-O	2.5	2.03	8.1	10.9
	Ru-Ru	4.3	2.66	8.9	3.8
RRCN-30	Ru-O	2.9	2.01	7.9	8.6
	Ru-Ru	3.8	2.67	9.2	4.0
RRCN-50	Ru-O	3.1	2.01	7.5	9.8
	Ru-Ru	3.3	2.66	9.8	3.5
RRCN-100	Ru-O	3.4	1.99	7.1	9.1
	Ru-Ru	3.1	2.67	9.5	4.4

CN, coordination number; *R*, bonding distance; σ^2, Debye-Waller factor; ΔE_0, inner potential shift.

Supplementary Table 2. Fitting parameters for TA kinetics of CN, RCN and RRCN.

		**CN**	**RCN**	**RRCN**
**1**	τ_1 (ps)	80	80	80
	A_1 (%)	100	54.1	0.1
**2**	τ_2 (ps)	/	1.73	1.73
	A_2 (%)	/	45.9	0.3
**3**	τ_3 (ps)	/	/	~0.1
	A_3 (%)	/	/	99.6

Supplementary Table 3. Energy levels of CN, RCN and RRCN samples calculated from UPS and UV-vis spectra data.

Sample	E_L (eV)	E_H (eV)	E_g (eV)	E_{VBM} (eV)	E_{CBM} (eV)
CN	2.36	17.29	2.96	6.29	3.33
RCN	2.47	17.77	3.03	5.92	2.89
RRCN	2.34	17.64	3.01	5.92	2.91

4. References

1. Newville, M. IFEFFIT: interactive XAFS analysis and FEFF fitting IFEFFI. *J. Synchrotron Radiat.* **8**, 322-324 (2001).
2. Ravel, B. N., M. ATHENA, ARTEMIS, HEPHAESTUS: data analysis for X-ray absorption spectroscopy using IFEFFIT. *J Synchrotron Radiat* **12**, 537-541 (2005).
3. Ankudinov, A. L. *et al.* Real-space multiple-scattering calculation and interpretation of x-ray-absorption near-edge structure. *Phys. Rev. B* **58**, 7565-7576 (1998).
4. Huang, J. *et al.* Oxyhydroxide Nanosheets with Highly Efficient Electron-Hole Pair Separation for Hydrogen Evolution. *Angew. Chem. Int. Ed.* **55**, 2137-2141 (2016).
